# *TP53* mutations drive therapy resistance through post-mitochondrial caspase blockade

**DOI:** 10.1038/s41419-026-09087-0

**Published:** 2026-07-28

**Authors:** Ahmed M. Mamdouh, Fizza Iftikhar, Elyse A. Olesinski, Fang Qi Lim, Shaista Jasdanwala, Yuhan Wang, Yang Mi, Nicole Si En Lin, Daniel Tan En Liang, Md Al Nayem Chowdhury, Nishtha Chitkara, Leah Hogdal, Coleman R. Lindsley, Anthony Letai, Koji Itahana, Shruti Bhatt

**Affiliations:** 1 Department of Pharmacy and Pharmaceutical Sciences, National University of Singapore, Singapore, Singapore; 2 Department of Hematology & Medical Oncology, Emory School of Medicine and the Winship Cancer Institute, Emory University, Atlanta, GA USA; 3 Department of Medicine, Massachusetts General Hospital, Boston, MA USA; 4 Dana-Farber Cancer Institute, 440 Brookline Avenue, M430 Boston, MA USA; 5 Program in Cancer & Stem Cell Biology, Duke-NUS Medical School, Singapore, Singapore

**Keywords:** Acute myeloid leukaemia, Molecular biology

## Abstract

Acute myeloid leukemia (AML) is a heterogeneous disease characterized by a broad spectrum of molecular alterations that influence clinical outcomes. *TP53* mutations define one of the most lethal subtypes of AML, driving resistance to nearly all available treatment modalities, including venetoclax plus azacitidine (VenAza). Yet, the molecular basis of this resistance, beyond affecting transactivation of BCL-2 family genes, has remained elusive. Here, we demonstrate that VenAza treatment leads to reduced transcriptional upregulation of the p53 signaling pathway in *TP53* mutant/deficient AML compared to wild-type AML. Functionally, *TP53* mutant/deficient AML exhibits selective failure in apoptosis induction rather than impaired G1 arrest or senescence. Despite inhibition of pro-apoptotic BAX and selective enrichment for MCL-1 in *TP53* mutant isogenic AML cells, compensatory upregulation of BIM preserved functional mitochondrial outer membrane permeabilization (MOMP). *TP53* mutant primary AML tumors at baseline also had retained capacity for MOMP. Instead, *TP53* mutant AML exhibited disruption in caspase-3/7 activation to evade apoptosis after VenAza therapy – decoupling the mitochondrial and executioner phases of apoptosis. Importantly, this “post-MOMP brake” is not a bystander effect but itself a driver of VenAza and chemotherapy resistance in *TP53* mutant/deficient AML. This previously unrecognized mechanistic insight shifts the focus from mitochondrial priming to terminal caspase blockade in *TP53* mutant AML and opens the door for urgently needed therapeutic strategies that reignite apoptosis at its execution point.

## Introduction

*TP53* mutations or deletions are well-established drivers of therapy resistance across many cancers, leading to poor patient outcomes following chemotherapy and targeted therapy [1–3]. In acute myeloid leukemia (AML), an aggressive hematological malignancy, *TP53* alterations confer particularly dismal prognoses. These mutations occur in ~10% of de novo AML cases, 20-35% of therapy-related myeloid neoplasia, and up to 70-80% of complex karyotype AML (CK-AML) [4–6]. The advent of venetoclax (BCL-2 inhibitor) combined with azacitidine (hypomethylating agent) (VenAza) has transformed outcomes for many AML patients [7], including those with poor-risk cytogenetics and wild-type *TP53*, who traditionally responded poorly to 7 + 3 intensive chemotherapy [8–11]. However, VenAza offers very little benefit to patients with *TP53* mutations, particularly when co-occurring with complex karyotypes. In this subgroup, median overall survival plummets from 23.4 months in wild-type cases to just 5.2 months in *TP53* mutant patients [7]. This stark disparity persists regardless of cytogenetic risk status, underscoring *TP53* mutations as an independent predictor of treatment failure. Patients harboring *TP53* mutations also experience significantly shorter relapse-free survival (4.3 months vs. 18.9 months) and overall survival (5.2 months vs. 19.4 months), emphasizing the urgent need for new therapeutic strategies [11–13].

The tumor suppressor p53 functions as the “guardian of the genome” by transactivating genes in response to stress, including those regulating genomic integrity, cell cycle control, and apoptosis induction [11, 14]. Canonically, p53 induces apoptosis primarily by triggering mitochondrial outer membrane permeabilization (MOMP) via transcriptional activation of pro-apoptotic BCL-2 family members (e.g. BAX, PUMA, and NOXA) [15, 16]. Supporting this, studies have shown that *TP53* deficient cells cannot activate BAX following venetoclax treatment to induce MOMP [17]. Additionally, p53 can be sequestered in the cytoplasm by anti-apoptotic BCL-2 proteins (e.g. MCL-1, BCL-2 and BCL-xL) and released by PUMA to promote MOMP [18–22]. While MOMP is considered the ‘point-of-no-return’ in apoptotic signaling, emerging studies suggests that even after MOMP, wild-type p53-expressing cells may recover [23], indicating the existence of additional regulatory steps downstream of mitochondrial permeabilization.

Despite p53’s central role in apoptosis, the precise mechanisms by which *TP53* mutations drive therapy resistance remain incompletely defined. Here, we identify among different functions of p53 that disruption in apoptosis is the leading contributor to VenAza resistance. One may expect evasion of apoptosis due to failure of MOMP induction in *TP53* mutant/deficient cells, but we show that MOMP remains proficient. Instead, *TP53* mutant AML escapes apoptosis via failure to activate post-mitochondrial executioner caspases. These novel findings highlight that *TP53* mutant AML effectively uncouples mitochondrial permeabilization from post-mitochondrial signaling to evade apoptosis.

## Materials and methods

### Cell lines

AML cell lines were obtained as follows: MOLM-13 from DSMZ, MV-4-11 from ATCC. MOLM-13-*TP53* isogenic AML cell lines *TP53*^*+/+*^ (wild-type), *TP53*^*-/-*^ (knock-out), and six mutants, *TP53*^*-/R248Q*^, *TP53*^*-/Y220C*^, *TP53*^*-/R282W*^, *TP53*^*-/R175H*^, *TP53*^*-/R273H*^, and *TP53*^*-/M237I*^ were kindly provided by Steffen Boettcher [24]. Lenti-X 293 T, and U2OS cell lines were kindly provided by Nicholas Gascoigne, and Karen Crasta, respectively. Isogenic MOLM-13 cells were cultured in RPMI-1640 (Gibco, 11875093). Lenti-X 293 T, and U2OS cells were cultured in DMEM (Biowest, L0104). Media was supplemented with 10% fetal bovine serum (Gibco, 10437028) and 1% penicillin-streptomycin (Gibco, 15140122). All cell lines were cultured in a humidified incubator at 37°C with 5% CO_2_.

### Generation of knockout cells

sgRNA sequences were cloned into lentiCRISPRv2 (Addgene, #52961). Lenti-X 293 T cells (1.8×10^6^ cells) were transfected with envelope plasmid pVSV-G (415 ng), packaging plasmid psPAX2 (830 ng), and the corresponding transfer plasmids (1245 ng) using Lipofectamine™ 3000 transfection reagent (ThermoFisher, L3000015) and Opti-MEM (Gibco, 31985070). Cell culture media was replaced with DMEM after 24 h. Supernatants containing lentiviruses were collected at 48 h and 72 h post transfection and filtered through a 0.45 μM nitrocellulose filter (Sartorius). AML cells were centrifuged at a speed of 2500 rpm for 90 min at 32°C with 200 μL of virus-containing media supplemented with 10 μg/mL polybrene (MedChemExpress). Puromycin (2 μg/mL) or hygromycin (400 µg/ml) was added 48 h post-infection. sgRNA sequences are listed in Supplemental Table 1.

### Alkaline comet assay

We pre-coated glass slides with 1% (w/v) agarose in type I grade water and dried them overnight at room temperature. For each treatment group, 1.0 ×10^5^ cells were resuspended in 100 µL fresh media and 200 μL of 1% (w/v) low-melting agarose in PBS. Samples were added to pre-coated slides and cooled at 4°C to solidify. Slides were treated with alkaline lysis buffer by immersion in an alkaline electrophoresis solution at 4°C for 40 min before electrophoresis was conducted at room temperature. Slides were neutralized, fixed, stained with propidium iodide, and rinsed with PBS. Images were collected on a Nikon SMZ25 fluorescence microscope. Quantification done using CometScore 2.0.

### Beta-galactosidase senescence assay

1.0×10^6^ cells were treated before analysis by flow cytometry according to the manufacturer’s protocol (Invitrogen). Cells were fixed in 4% paraformaldehyde for 10 min at room temperature. Subsequently, cells were stained with CellEvent Senescence Green Probe, diluted at a ratio of 1:2000 in CellEvent Senescence Buffer, and incubated at 37°C in the dark for 1 h. Data was acquired on Attune NxT Flow Cytometer (Invitrogen) and analyzed on FlowJo. For positive control, U2OS cells were treated with nocodazole (100 ng/mL) for 96 h.

### BH3 profiling and dynamic BH3 profiling

To determine priming for apoptosis, 2.5×10^4^ cells were resuspended into 15 µL of MEB buffer and seeded into a 384-well plate. BIM peptide (determinant for overall priming) in different concentrations was mixed with 0.04% digitonin and added into cells. DMSO and alamethicin were used as negative and positive controls, depicting 100% and 0% cytochrome c retention, respectively. Mitochondria of permeabilized cells were exposed to peptides for 1 h at room temperature with shaking and fixed in the dark with 4% paraformaldehyde for 10 min with shaking. Cells were subsequently neutralized with N2 buffer for 15 min with shaking and stained with Hoechst and anti-cytochrome c-Alexa647 (BioLegend, # 612310). At 24 h post-incubation, quantification of cytochrome c release induced by each peptide was analyzed using flow cytometry (FACSCelesta). Mitochondrial priming was measured as FACS-determined cytochrome c loss = 100 − (% of cells within cytochrome c retention gate) [25]. For dynamic BH3 profiling, cells were treated with the desired agent for 4 h before harvesting, after which samples were processed in the manner mentioned above. Drug-induced change in priming, or “delta priming,” was calculated as = cytochrome c loss [drug] – cytochrome c loss [DMSO].

### Caspase luminescence assay

1.0×10^4^ MOLM-13 cells were seeded in 100 µL RPMI per well in 96-well plates and treated with agents of interest. After 24 h of incubation at 37°C, 30 μL was aliquoted from each well and added to a 384-well plate. Next, 30 μL of Caspase Glo (Promega) was added and incubated for 1.5 h at room temperature. The luminescence intensity was measured by a Hidex sense multi-plate reader [24].

### TMRE staining

Cells were seeded at a density of 1.0×10^6^ cells per mL of media. The cells were stained with TMRE (10 nM) (Invitrogen) and incubated at 37°C with 5% CO_2_ for 30 min. Cells were pelleted before resuspending in fresh media and treated. Cells were further incubated for 24 h before staining with DAPI and analysis by flow cytometry.

### Statistical significance

Statistical analyses were performed using GraphPad Prism (version 10.0). All tests were two-sided. Comparisons between two groups were conducted using two-tailed t-tests unless otherwise specified. For experiments with multiple groups, two-way analysis of variance (ANOVA) was performed, followed by Šídák or Bonferroni correction for multiple comparisons, as indicated. Adjusted P values are reported where applicable. Data are presented as mean ± SEM; center values represent the mean. Significance levels are defined as *p < 0.05, **p < 0.01, ***p < 0.001, ****p < 0.0001.

## Results

### *TP53* mutation/deletion causes resistance to VenAza

**Fig. 1:**
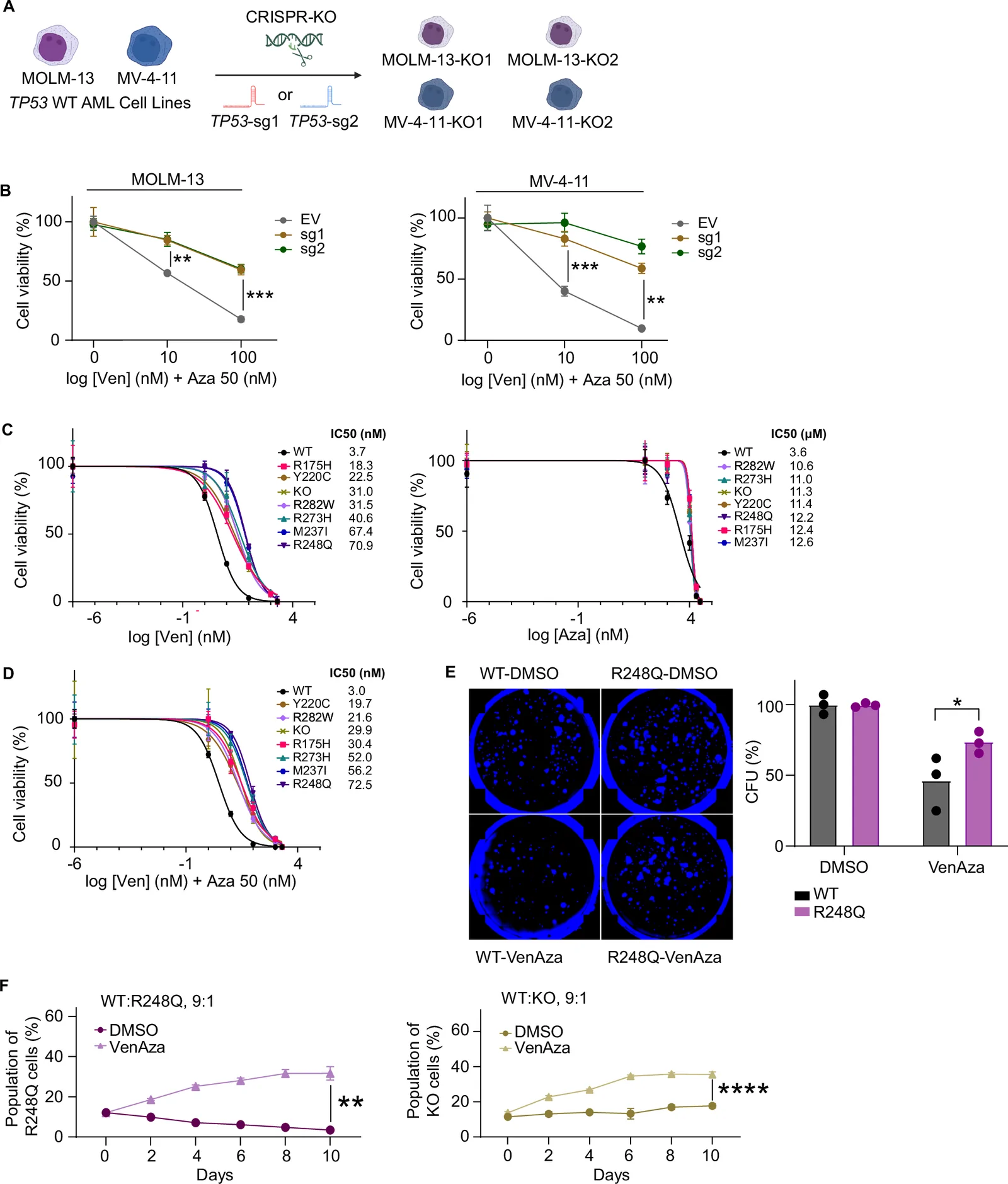
*TP53* mutant AML is resistant to VenAza. **A** Schematic of CRISPR-KO generation of *TP53* knockout MOLM-13. **B** Cell viability of *TP53* active (EV) and knockout (sg1, sg2) AML cells (MOLM-13, MV-4-11) treated with venetoclax and azacitidine. **C** Cell viability of isogenic MOLM-13 cells after 72 h of treatment with increasing concentrations of venetoclax or azacitidine as a single agent. **D** Cell viability of isogenic MOLM-13 cells after 72 h of treatment with increasing concentrations of venetoclax and azacitidine. **E** Representative images of colony formation assays after treatment with DMSO or VenAza for 72 h in RPMI followed by 9 days in Methocult. Colony-forming units were normalized to respective DMSO-treated cells. **F** Clonal competition assay evaluating the competition between the cells in co-culture after treatment with DMSO or VenAza for 10 days. For all figures unless otherwise specified, treatment with VenAza: venetoclax 3 nM, azacitidine 50 nM. *p < 0.05, **p < 0.01, ***p < 0.001, ****p < 0.0001.

Although the clinical challenge of therapy resistance in *TP53* mutant AML has been recognized, the underlying mechanisms remain incompletely understood. Hence, to study the mechanisms of resistance, we engineered *TP53-*deficient AML cells using CRISPR/Cas9 genome editing in MOLM-13 and MV-4-11 with two different sgRNAs (Figs. 1A and S1A). Successful knock-out was validated by evaluating the expression of *TP53* and *CDKN1A* (Fig. S1,B). *TP53* deletion reduced sensitivity to VenAza compared to wild-type *TP53* (Fig. 1B). Next, we exposed a panel of isogenic MOLM-13 cells [24] carrying six different missense mutations in the DNA-binding domain (DBD) (TP53^-/mut^: R175H, R273H, R248Q, R282W, Y220C, M237I), TP53^-/-^, or TP53^+/+^ to venetoclax-only or azacitidine-only. All *TP53*^*-/mut*^ and *TP53*^*-/-*^ had less sensitivity compared to *TP53*^*+/+*^ when treated with either venetoclax or azacitidine as a single agent, as shown before [26] (Fig. 1C). Functional inactivation of p53 signaling in mutant/null cells was confirmed by variable p53 expression (Fig. S1C) and treatment with the MDM2 inhibitor, nutlin-3A. *TP53* wild-type (*TP53*^*+/+*^) cells exhibited dose-dependent sensitivity to nutlin-3A, whereas *TP53*^*-/mut*^ and *TP53*^*-/-*^ were resistant (Fig. S1D).

To assess differential sensitivity among the six distinct TP53 missense mutations, we combined low-dose azacitidine (50 nM) with venetoclax. *TP53*^*-/R248Q*^ cells showed the least sensitivity compared to other missense mutations (IC50 for VenAza combination = 72.5 nM; Fig. 1D). *R248Q* mutation is the second most prevalent *TP53* mutation among other missense mutations in myeloid malignancies [24], Hence, we decided to focus on the *R248Q* mutation in subsequent experiments. *TP53*^*-/R248Q*^ cells had higher colony forming units (CFUs) under VenAza compared to *TP53*^*+/+*^ (76.9% vs. 56.4%; Fig. 1E). Further validation in an additional isogenic cell line *TP53*^*-/-*^ MV-4-11 showed significantly increased clonogenic survival following VenAza treatment relative to matched *TP53*^*+/+*^ controls (Fig. S1E). All six *TP53*^*-/mut*^ and *TP53*^*-/-*^ cell lines had a competitive advantage over *TP53*^*+/+*^ cells treated with VenAza (Figs. 1F and S1F). In conclusion, *TP53* mutation or deletion caused resistance to venetoclax alone, azacitidine alone, and VenAza combination.

### *TP53* mutations impair VenAza-induced p53 signaling and associated transcriptional programs

**Fig. 2:**
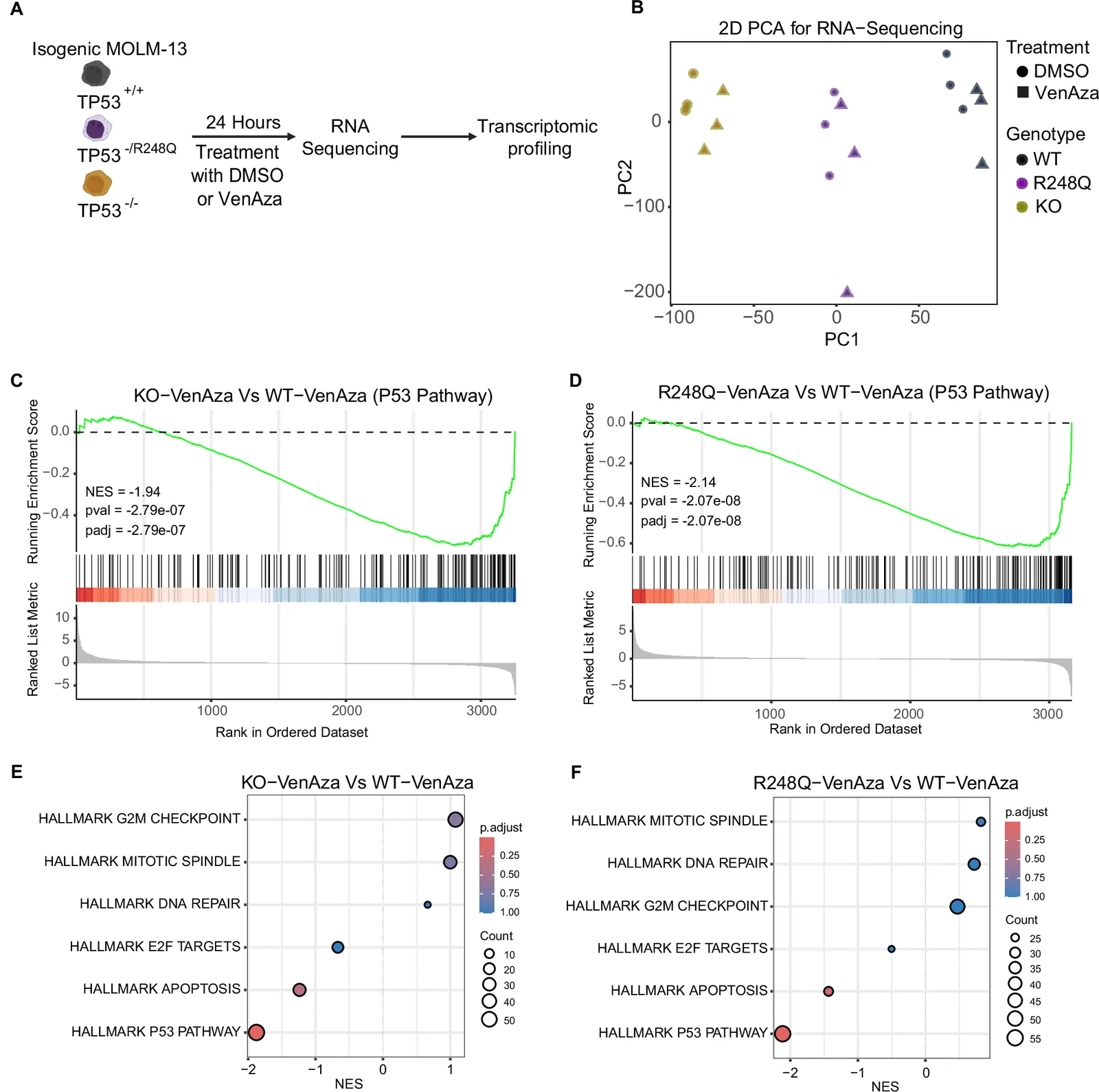
Transcriptomic profiles of *TP53* mutant AML. **A** Schematic overview of the experimental design of RNA sequencing. Isogenic MOLM-13 cells were treated with DMSO or VenAza for 24 h, followed by RNA-seq for transcriptomic profiling. **B** Principal component analysis (PCA) of RNA-seq data from all samples. PC1 and PC2 distinguish samples based on genotype and treatment. Genotypes are color-coded, while treatment conditions are indicated by shape. **C–F** Gene set enrichment analysis (GSEA) using MsigDB for 6 hallmarks related to the main p53 canonical functions: **C** p53 pathway for *TP53*^*-/-*^ treated with VenAza vs. *TP53*^+/+^ treated with VenAza; **D** p53 pathway for *TP53*^*-/R248Q*^ treated with VenAza vs. *TP53*^+/+^ treated with VenAza; **E** other pathways for *TP53*^*-/-*^ treated with VenAza vs. *TP53*^+/+^ treated with VenAza; **F** other pathways for *TP53*^*-/R248Q*^ treated with VenAza vs. *TP53*^+/+^ treated with VenAza. For all figures unless otherwise specified, treatment with VenAza: venetoclax 3 nM, azacitidine 50 nM.

Since p53 coordinates key stress response pathways, we investigated whether VenAza response is affected in *TP53* genotypes via transcriptional regulation of its canonical functions – DNA damage response, cell cycle arrest, and apoptosis – which has previously been evaluated in *TP53*^*-/-*^ lymphoma [27] but not AML. To this end, we performed RNA sequencing on isogenic MOLM-13 cells harboring *TP53*^*+/+*^, *TP53*^*-/R248Q*^, or *TP53*^*-/-*^ alleles following treatment with VenAza or DMSO as a control (Fig. 2A). Principal component analysis (PCA) revealed clear segregation of transcriptional signatures by *TP53* genotype (Figs. 2B and S2A). Further, VenAza exposure also modulated distinct transcriptional programs within each genotype.

We next conducted gene set enrichment analysis (GSEA) using the MsigDB Hallmark collection to evaluate gene sets for the p53 pathway, DNA repair, apoptosis, and cell cycle (E2F targets for G1-S phase, G2M checkpoint, and mitotic spindle). As anticipated, p53 signaling was down-regulated in *TP53*^*-/R248Q*^ and *TP53*^*-/-*^ cells at baseline compared to *TP53*^*+/+*^ cells (*p* < 0.0001; Fig. S2B, C). We then asked whether VenAza exposure influenced p53 signaling in a genotype-specific manner. Compared to DMSO, VenAza led to significant enrichment in p53 pathways for *TP53*^*+/+*^ (*p* < 0.0001) and *TP53*^*-/R248Q*^ (*p* < 0.05) cells but not *TP53*^*-/-*^ (*p* > 0.05) cells (Fig. S2,D–F). Remarkably, the induction of p53 signaling was significantly suppressed in *TP53*^*-/R248Q*^ and *TP53*^*-/-*^ cells compared to *TP53*^*+/+*^ cells following VenAza treatment (*p* < 0.0001; Fig. 2C, D). Among other p53-regulated canonical pathways, the apoptosis pathway was modestly downregulated, while DNA repair, G2M, and mitotic spindle pathways were modestly upregulated (*p* > 0.05; Fig. 2E, F). Together, this indicates that intact p53 signaling in AML in response to VenAza is upregulated in *TP53*^*+/+*^ cells compared to *TP53*^*-/R248Q*^ and *TP53*^*-/-*^ cells. Nevertheless, programs involved in cell cycle progression and DNA repair are less affected transcriptionally in response to VenAza.

### *TP53* mutations selectively impair VenAza-induced apoptosis without affecting cell cycle progression or senescence

**Fig. 3:**
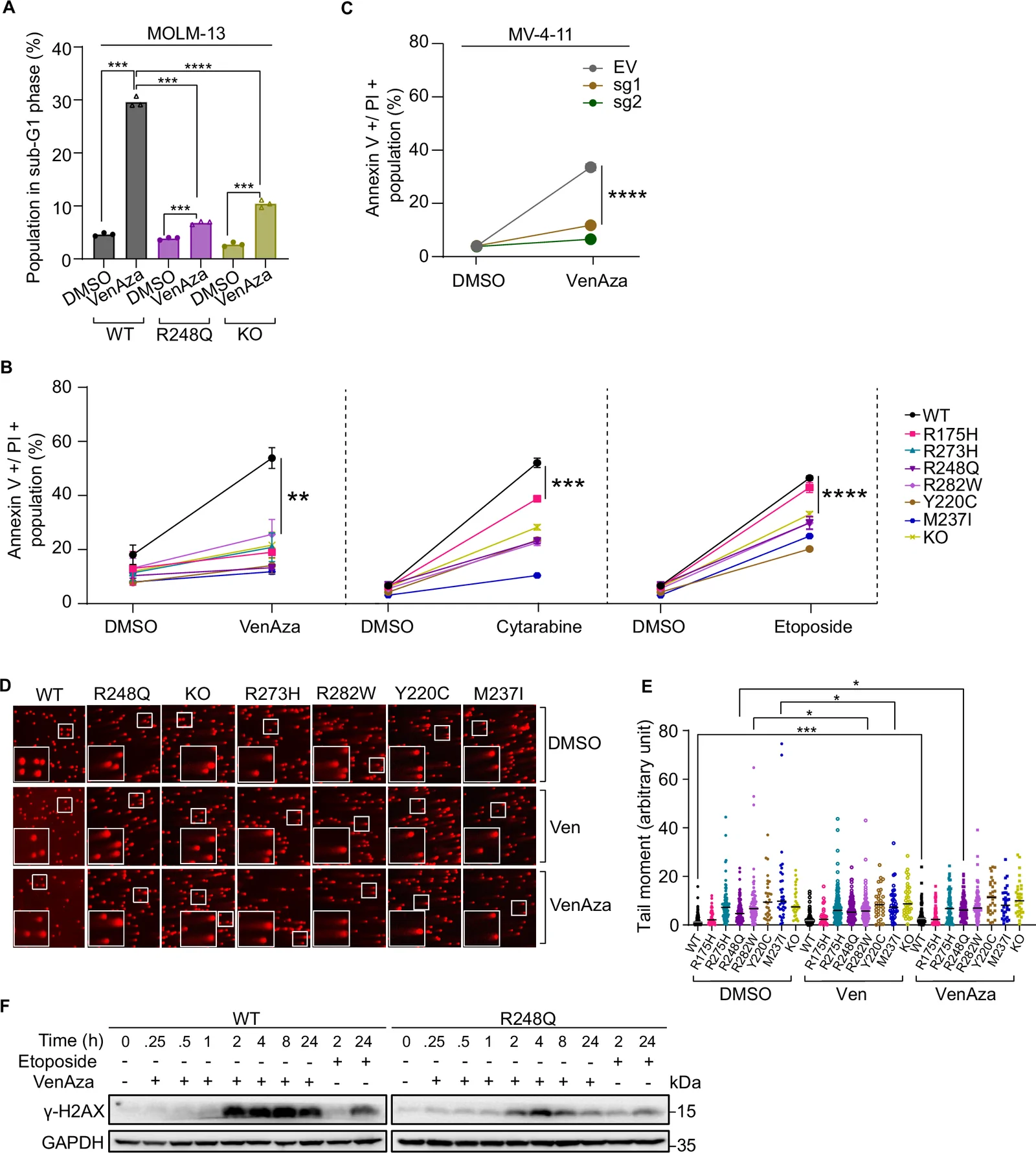
*TP53* mutant AML resists VenAza treatment by evasion of apoptosis. **A** Population of cells in sub-G1 phase MOLM-13 *TP53*^+/+^, *TP53*^-/R248Q^, and *TP53*^*-/-*^ cells treated with DMSO or VenAza for 24 h. **B** Annexin V and PI assay demonstrate greater viable cell populations in *TP53*^*-/mut*^ and *TP53*^*-/-*^ isogenic MOLM-13 cells treated with VenAza, cytarabine (200 nM), or etoposide (200 nM) for 72 h. **C** Annexin V and PI assay for MV-4-11 after treatment with venetoclax 10 nM plus azacitidine 50 nM for 72 h. **D** Alkaline comet assay of isogenic *TP53*^*+/+*^*, TP53*^*-/mut*^, *TP53*^*-/-*^ cells MOLM-13 cells treated with DMSO, venetoclax (1 nM), or VenAza (venetoclax 1 nM, azacitidine 50 nM) for 7 days. **E** Quantification of comet tails normalized to *TP53*^+/+^ DMSO. **F** Time course western blot of γ-H2AX after treatment with VenAza or etoposide (0.5 μM). For all figures unless otherwise specified, treatment with VenAza: venetoclax 3 nM, azacitidine 50 nM. *p < 0.05, **p < 0.01, ***p < 0.001, ****p < 0.0001.

After transcriptomic profiling, we next investigated whether VenAza treatment functionally affects *TP53*’s main canonical roles in cell cycle regulation, apoptosis, senescence, and DNA damage repair. To test this, we treated isogenic MOLM-13 cells carrying six different *TP53*^*-/mut*^ cells, *TP53*^*-/-*^ cells, or matched *TP53*^*+/+*^ cells with VenAza. At baseline, *TP53*^*-/R248Q*^ and *TP53*^*-/-*^ cells showed no major differences in cell cycle profiles or senescence compared to *TP53*^*+/+*^ cells (Fig. 3A and S3A, B). However, following VenAza, *TP53*^*-/R248Q*^ and *TP53*^*-/-*^ cells failed to accumulate in the sub-G1 phase, indicating defective apoptosis despite intact mitotic entry that supported transcriptomic changes. Apoptosis was severely blunted across all *TP53*^*-/mut*^ and *TP53*^*-/-*^ MOLM-13 cells, as well as in isogenic TP53^-/-^ MV-4-11 cells treated with VenAza, in response to both VenAza and standard chemotherapy (cytarabine or etoposide) (Fig. 3B, C).

To assess whether this apoptotic defect was linked to DNA damage, we performed alkaline comet assays. In *TP53*^*+/+*^ cells, VenAza increased DNA damage relative to DMSO (Fig. 3D, E). In contrast, *TP53*^*-/mut*^ cells displayed elevated baseline DNA damage that did not further increase with treatment. We also assessed γ-H2AX levels as a marker of double-strand DNA breaks. *TP53*^*+/+*^ cells showed rapid induction of γ-H2AX following treatment, with levels surpassing those observed in *TP53*^*-/R248Q*^ cells at both early and late time points (Fig. 3F). These results suggest that *TP53* mutations drive resistance to cytotoxic and targeted therapies in AML by disrupting apoptosis and resisting DNA damage effects, rather than through disruption of senescence or cell cycle signaling.

### *TP53* mutations paradoxically upregulate BIM while enhancing its sequestration by MCL-1

**Fig. 4:**
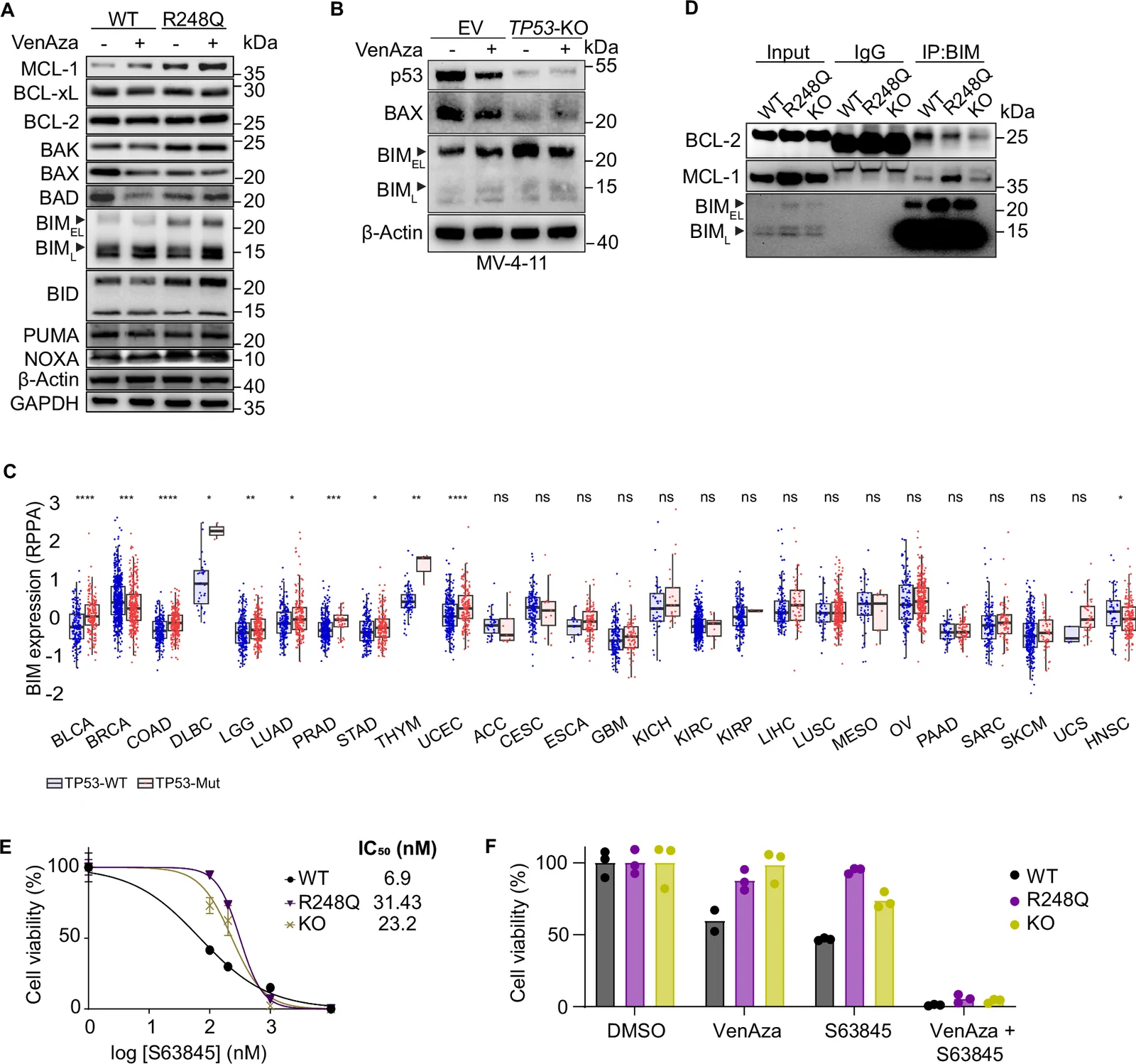
*TP53* mutations alter BCL-2 family proteins and respond to simultaneous BCL-2/MCL-1 targeting. **A** Immunoblotting of BH3 family proteins and other apoptotic factors in *TP53*^*+/+*^ and *TP53*^*-/R248Q*^ cells treated with DMSO or VenAza for 24 h. **B** Western blot of EV and *TP53*^*-/-*^ MV-4-11 cells treated with DMSO or VenAza (venetoclax 10 nM, azacitidine 50 nM) for 24 h. **C** BIM protein expression levels across multiple cancers from the TCGA database, stratified according to *TP53* status. Analysis via Wilcoxon test. **D** Coimmunoprecipitation assay identifies interactions between BIM:MCL-1 or BIM:BCL-2. **E** Cell viability of MOLM-13 isogenic cells after exposure to **E** S63845 for 72 h or **F** DMSO, VenAza, S63845 (10 nM), or VenAza plus S63845 (10 nM) for 72 h. For all figures unless otherwise specified, treatment with VenAza: venetoclax 3 nM, azacitidine 50 nM. *p < 0.05, **p < 0.01, ***p < 0.001, ****p < 0.0001.

To investigate mechanisms of apoptotic resistance, we next examined expression of BCL-2 family proteins – key regulators of the intrinsic mitochondrial pathway and therapeutic targets of venetoclax. As expected, *TP53* target genes *CDKN1A* and *MDM2* transcripts were downregulated in *TP53*^*-/R248Q*^ and *TP53*^*-/-*^ cells at baseline (Fig. S4A). Since *TP53* regulates apoptotic genes, we hypothesized that VenAza would alter expression of BCL-2 family genes and proteins in a *TP53*-dependent manner. At the mRNA level, pro-apoptotic genes *BAX*, *BBC3* (encodes PUMA), and *PMAIP1* (encodes NOXA) were downregulated in *TP53*^*-/R248Q*^ cells compared to *TP53*^*+/+*^ cells at the baseline, as well as in the presence of VenAza (Fig. S4,A). At the protein level, pro-apoptotic protein BAX was decreased, and the anti-apoptotic protein MCL-1 was increased (Fig. 4A and S4B). Other studies have implicated increased MCL-1 as a potential mediator of BIM upregulation [27, 28]. Conversely, pro-apoptotic proteins BIM-EL, BAK, and BID were upregulated. Since BIM can directly interact with and activate BAX and BAK (required for pore formation and MOMP), we further investigated BIM expression levels across multiple tumors. MV-4-11 cells showed upregulation in BIM-EL after *TP53* deletion (Fig. 4B and S4C). Similarly, primary tumors from the TCGA Pan-Cancer Atlas showed upregulation in BIM for 5/27 tumors at the mRNA level and 10/26 at the protein level (Fig. 4C and S4D). This emphasizes that despite loss of key pro-apoptotic regulators in *TP53* mutations, upregulation of BIM and BID may compensate for this downregulation.

Sequestration of BIM by anti-apoptotic BCL-2 family members can inhibit apoptosis. Prior studies, including ours, have shown that in venetoclax-resistant AML, MCL-1 sequesters BIM away from BCL-2 [29–31]. Coimmunoprecipitation assays revealed reduced BCL-2–BIM interactions and increased MCL-1–BIM interactions in these cells (Fig. 4D and S4E). Although *TP53*^*-/R248Q*^ and *TP53*^*-/-*^ cells were more resistant to MCL-1 inhibitor S63845, combining the MCL-1 inhibitor with VenAza restored sensitivity in *TP53* mutant cells, consistent with prior reports [32] (Fig. 4E, F). However, while targeting MCL-1 is a rational approach to overcome resistance, clinical development has been hampered by dose-limiting cardiotoxicity [33]. Together, *TP53* mutations reprogram both expression and functional interactions of BCL-2 family proteins.

### MOMP occurs in *TP53* mutant AML despite alterations in BCL-2 family proteins

**Fig. 5:**
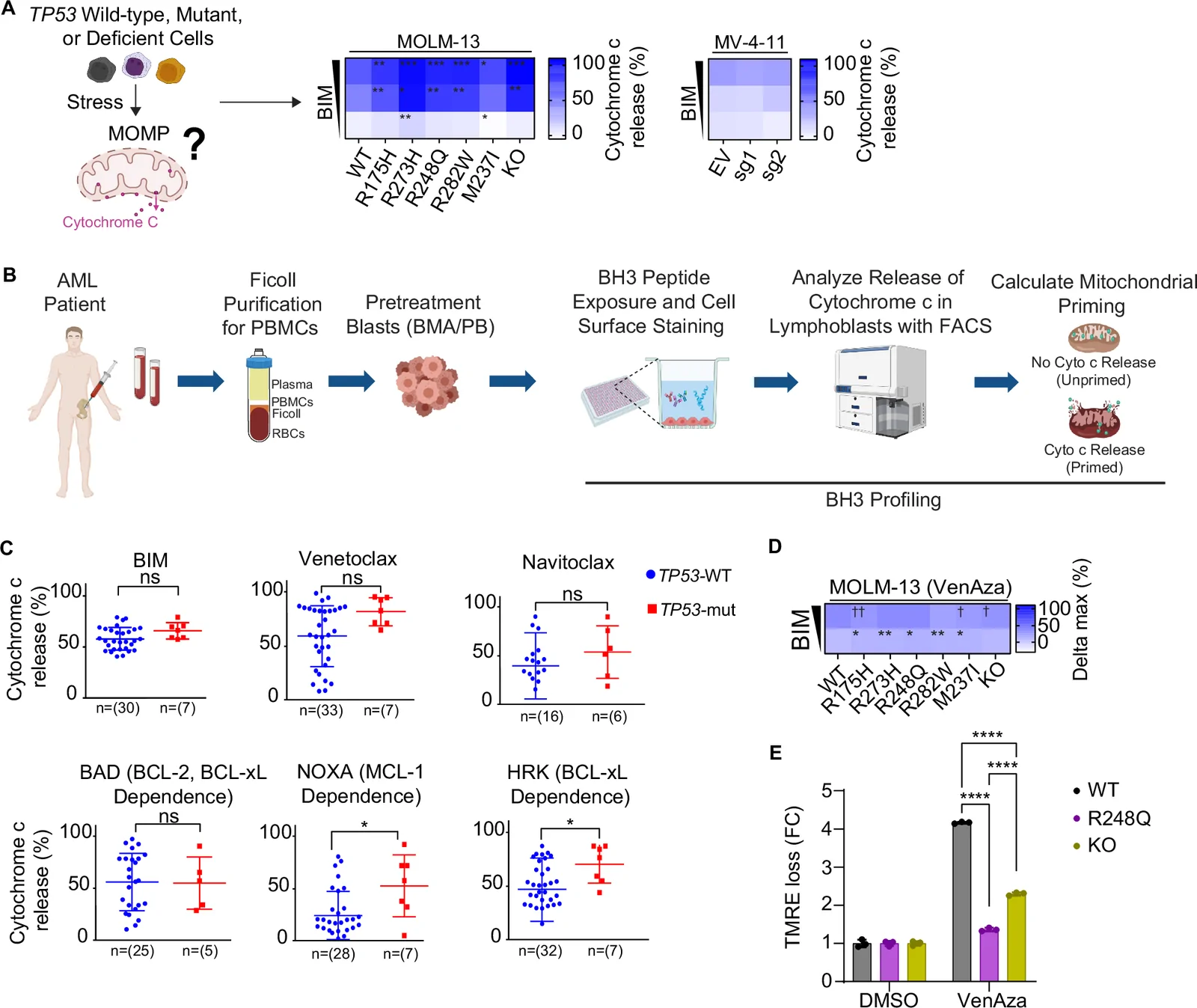
*TP53* mutant AML maintains the potential to undergo MOMP. **A** Baseline BH3 profiling heatmap shows mitochondrial priming response of DMSO and titrated BIM for AML (MOLM-13 *TP53*^+/+^*, TP53*^-/mut^, *TP53*^-/-^, and MV-4-11). BIM concentrations used: MOLM-13 *TP53*^+/+^*, TP53*^-/mut^, *TP53*^-/-^: 0.03-3.0 μM; MV-4-11: 0.1-0.5 μM. **B** Experimental schematic of the baseline BH3 profiling for primary AML samples. **C** BH3 profiling for primary AML samples based on *TP53* status using different peptides and drugs as peptides. **D** Dynamic BH3 profiling heatmap after treating MOLM-13 isogenic *TP53*^*+/+*^*, TP53*^*-/mut*^, *TP53*^*-/-*^ cells for 4 h with VenAza (1 nM venetoclax and 50 nM azacitidine). Dagger = decreased priming. Asterisk = increased priming. **E** TMRE loss for *TP53*^+/+^, *TP53*^-/R248Q^, and *TP53*^*-/-*^ cells treated with DMSO or VenAza (venetoclax 3 nM and azacitidine 50 nM) for 24 h; Analysis via two-way ANOVA followed by Tukey. *p < 0.05, **p < 0.01, ***p < 0.001, ****p < 0.0001.

Apoptosis critically depends on MOMP, a key step in the intrinsic apoptotic pathway often considered the ‘point of no return.’ Thus, we hypothesized that *TP53* mutant cells evade apoptosis by resisting MOMP induction. We first measured baseline capacity for MOMP in unperturbed permeabilized cells using BH3 peptides derived from pro-apoptotic proteins. Across multiple contexts – *TP53*^*+/+*^, *TP53*^*-/mut*^, and *TP53*^*-/-*^ isogenic MOLM-13 cells and *TP53*^*-/-*^ MOLM-13 and MV-4-11 edited with distinct *TP53* sgRNAs – we consistently observed retained capacity for MOMP induction in response to the BIM BH3 peptide (Fig. 5A and S5A). These findings suggest that *TP53* mutant and deficient cells maintain intact apoptotic priming. BH3 profiling revealed no change in individual anti-apoptotic proteins – BCL-2, (BAD BH3 peptide), MCL-1 (MS-1 BH3 peptide), or BCL-xL (HRK BH3 peptide) – in *TP53*^*-/mut*^ vs. *TP53*^*+/+*^ cells (Fig. S5,B). Further validation in AML primary tumors showed comparable BIM-induced apoptotic priming across *TP53* genotypes and preserved direct mitochondrial sensitivity to navitoclax and venetoclax despite dysfunction in *TP53* (Fig. 5B, C). Increased dependence on BCL-xL and MCL-1 was also observed, as indicated by response to HRK and NOXA peptides, respectively. We confirmed that preserved MOMP in *TP53* mutant cells was not attributable to differences in baseline cytoplasmic cytochrome c levels (Fig. S5,C).

To determine whether *TP53* mutant cells resist VenAza-induced MOMP, we performed dynamic BH3 profiling, which showed comparable MOMP induction in response to BIM BH3 peptide in *TP53*^*+/+*^ and *TP53*^*-/mut*^ cells after VenAza exposure (Fig. 5D). To directly evaluate MOMP induced by VenAza therapy, we next measured cytochrome c loss using flow cytometry and immunofluorescence imaging in TP53 isogenic MOLM-13 and MV-4-11 cells. Flow cytometric analysis demonstrated comparable cytochrome c release in *TP53*^+/+^ and *TP53*^*-/mut*^ cells after VenAza (Fig. S5,D). These findings were further supported by immunofluorescence imaging, which confirmed comparable cytochrome c release in *TP53*^*-/R248Q*^ and *TP53*^*-/-*^ cells following VenAza exposure (Fig. S5,E).

While cytochrome c release confirmed equivalent MOMP across *TP53* genotypes, we sought to corroborate these findings using an orthogonal measure of MOMP. We quantified cytosolic mitochondrial DNA (mtDNA) release following VenAza treatment, as mtDNA egress into the cytosol occurs downstream of BAX/BAK-mediated pore formation and is independent of caspase activity, providing a direct structural readout of MOMP. VenAza increased cytosolic mtDNA levels in both *TP53*^*+/+*^ and *TP53*^*-/mut*^ cells, consistent with induction of MOMP (Fig. S5,F). mtDNA release was abrogated in BAX/BAK-deficient cells, confirming that this effect is MOMP-dependent. Validation of cytosolic nuclear DNA (RPP30) levels demonstrating fraction purity and excluding nonspecific nuclear leakage is shown in Fig. S5,G. Having confirmed that MOMP proceeds equivalently regardless of TP53 status, we next asked whether the downstream consequences of MOMP, specifically, mitochondrial membrane potential (MMP) dissipation, were similarly preserved. *TP53*^*-/R248Q*^ and *TP53*^*-/-*^ cells had less MMP loss compared to *TP53*^*+/+*^ cells, measured using tetramethylrhodamine ethyl ester (TMRE) fluorescence (Fig. 5E). Unlike cytochrome c release and mtDNA egress, MMP loss during apoptosis is not solely a consequence of MOMP itself but is amplified by effector caspase activity in a feed-forward loop; when downstream caspase activation is impaired, MMP can remain paradoxically intact despite upstream MOMP having occurred [34, 35]. Collectively, these findings suggest that *TP53* mutant AML cells retain MOMP capacity after direct stress on the mitochondria, and apoptotic failure instead stems from defects of post-mitochondrial permeabilization.

### Impaired caspase activation inhibits drug-induced apoptosis in *TP53* mutant cells

**Fig. 6:**
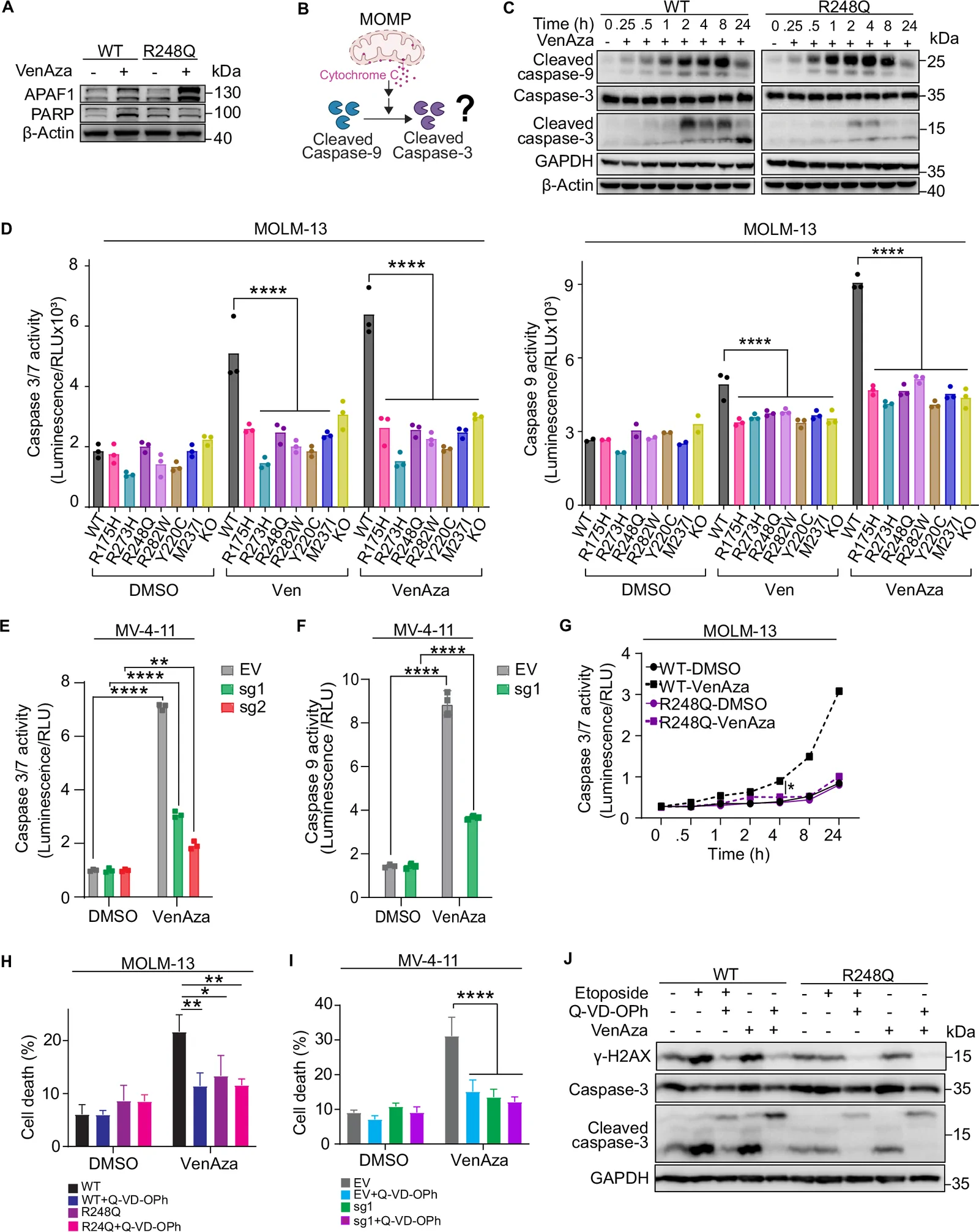
*TP53* mutant AML fails to activate caspases despite MOMP induction in response to therapy. **A** Western blot of Apaf-1 and PARP after treatment of MOLM-13 *TP53*^+/+^ and *TP53*^-/R248Q^ with VenAza for 24 h. **B** Schematic question of whether caspase activation occurs after MOMP induction in *TP53* mutant cells. **C** Western blot of cleaved caspase-9, cleaved caspase-3, and total caspase-3 after treatment of MOLM-13 *TP53*^+/+^ and *TP53*^-/R248Q^ with VenAza across different timepoints. Both plots developed simultaneously. **D** Caspase-3/7 and caspase-9 activity measured in isogenic MOLM-13 *TP53*^*+/+*^*, TP53*^*-/mut*^, *TP53*^*-/-*^ cells after 24 h of VenAza. Analysis via two-way ANOVA followed by Tukey. **E** Caspase-3/7 activity in MV4-11 EV and sg1 cells following 24-h treatment with DMSO or VenAza (venetoclax 10 nM, azacitidine 50 nM). **F** Caspase-9 activity was significantly reduced in response to VenAza in TP53-deficient MV4-11 cells as compared to WT, indicating impaired executioner caspase activation despite treatment. Analysis via two-way ANOVA followed by Tukey. **G** Caspase-3/7 activity in *TP53*^+/+^ and *TP53*^-/R248Q^ cells treated with DMSO or VenAza (venetoclax 1 nM, azacitidine 50 nM) across 24 h. **H** Percentage of cell death in MOLM-13 *TP53*^*+/+*^ and *TP53*^*R248Q*^ cells treated with DMSO or VenAza in the presence or absence of the pan-caspase inhibitor Q-VD-OPh for 24 h. Cell death was quantified by flow cytometry. Data were analyzed using two-way ANOVA with Tukey’s post hoc multiple comparisons test. **I** MV4-11 EV and sg1 cells were exposed to DMSO or VenAza for 24 h with or without Q-VD-OPh, followed by quantification of cell death (%). Apoptotic cells were assessed by Annexin V/PI staining and analyzed by flow cytometry. Data were analyzed by two-way ANOVA followed by Tukey’s multiple comparisons test. **J** Western blot of γ-H2AX, caspase-3, and cleaved caspase-3 after pre-treatment with Q-VD-OPh 25 mM for 4 h and then diluted to 5 mM for 24 h; treatment with VenAza or etoposide (0.5 μM) for 24 h. For all figures unless otherwise specified, treatment with VenAza: venetoclax 3 nM, azacitidine 50 nM. For all Figures Bars represent mean ± SEM. Adjusted *P* values are indicated as *p < 0.05; **p < 0.01; ****p < 0.0001; ns.

To identify the mechanism that restores apoptotic sensitivity for *TP53* mutant cells, we turned to post-mitochondrial regulators. Apaf-1 was upregulated in response to VenAza in *TP53*^*+/+*^ and *TP53*^*-/R248Q*^ cells, while PARP was cleaved only in *TP53*^*+/+*^(Fig. 6A). We then focused on the activation of initiator and executioner caspases to pinpoint where the apoptotic signaling is disrupted (Fig. 6B). Time-course analysis following VenAza treatment showed similar cleavage of initiator caspase-9 in *TP53*^*+/+*^ and *TP53*^*-/R248Q*^ *MOLM-13* cells and *TP53*^*-/-*^ MV-4-11 cells (Fig. 6C and S6A–C). However, executioner caspase-3 was cleaved selectively in *TP53*^*+/+*^ cells, revealing a disconnect between caspase-9 processing and downstream effector activation in the *TP53*^*-/mut*^ and *TP53*^*-/-*^ cells. Consistently, caspase-9 and -3/7 enzymatic activity was markedly diminished across all six isogenic *TP53*^*-/mut*^ and *TP53*^*-/-*^ MOLM-13 cells and *TP53*^*-/-*^ (Fig. 6D–F and S6D, E). We reasoned that although caspase-9 is cleaved, its enzymatic function is impaired in *TP53* mutant cells, preventing activation of caspase-3 and downstream apoptosis [34, 36]. Supporting this, VenAza triggered a time-dependent rise in caspase-3/7 activity in *TP53*^*+/+*^ cells but not in *TP53*^*-/R248Q*^ cells (Fig. 6G). We also assessed whether reduced caspase-3/7 activation in *TP53*^*-/R248Q*^ and *TP53*^*-/-*^ cells results from defects in the extrinsic apoptotic pathway. Although caspase-8 activity was modestly decreased in these cells, the reduction was far less pronounced than the observed loss for caspase-3/7 activity, suggesting that intrinsic pathway dysfunction is the primary driver of apoptotic failure (Fig. S6F).

To directly test whether VenAza-induced cell death is caspase-dependent, we treated *TP53*^*+/+*^, *TP53*^*-/mut*^ and *TP53*^*-/-*^ MOLM-13 and MV-4-11 AML cells with VenAza in the presence or absence of the pan-caspase inhibitor Q-VD-OPh and assessed apoptosis at 24 h. Caspase inhibition significantly reduced VenAza-induced cell death in *TP53*^*+/+*^cells to levels comparable to *TP53*^*-/mut*^ cells, effectively phenocopying the *TP53* deficient state (Fig. 6H and I). In contrast, *TP53*^*-/mut*^ cells, which already exhibit impaired apoptotic execution, showed minimal additional reduction in cell death upon Q-VD-OPh treatment. These findings further support that defective caspase activation underlies the resistance phenotype in *TP53* mutant AML.

Having established that post-MOMP caspase activation is impaired in TP53-mutant and knockout cells, we next investigated a downstream consequence of effector caspase activity: caspase-activated DNase (CAD)-mediated DNA fragmentation. During apoptosis, activated caspase-3 cleaves and releases CAD from its inhibitory chaperone ICAD, enabling CAD to translocate to the nucleus and induce internucleosomal DNA cleavage — a hallmark of apoptotic cell death. This caspase-dependent DNA damage is mechanistically distinct from genotoxic stress-induced DNA damage that activates the intrinsic pathway upstream of MOMP. We therefore hypothesized that the γ-H2AX signal observed following VenAza treatment reflects caspase-dependent, CAD-mediated DNA fragmentation rather than a primary DNA damage response, and that this signal would consequently be absent or attenuated in TP53-mutant cells where effector caspase activation is defective. To test this, we pre-treated cells with the pan-caspase inhibitor Q-VD-OPh, followed by VenAza exposure. Etoposide served as a positive control for DNA damage. Caspase inhibition completely abolished γ-H2AX induction in both *TP53*^*+/+*^ and *TP53*^*-/R248Q*^ cells, indicating that DNA damage resulting from VenAza is downstream of caspase activation (Fig. 6J). Genetic ablation of BAX/BAK markedly attenuated VenAza-induced γ-H2AX compared to control cells, confirming that γ-H2AX induction is post-MOMP (Fig. S6,G, H). Together, these findings suggest that functional *TP53* is required for full activation of the caspase cascade – particularly the link between caspase-9 and downstream executioner caspases – and is therefore critical for mounting an effective apoptotic response to VenAza in AML.

## Discussion

Therapy resistance remains a formidable barrier to curative treatment in *TP53* mutant cancers, particularly in AML, where patients frequently exhibit primary refractoriness to both conventional chemotherapy and standard of care VenAza [1–3, 8–11]. Clinical and pre-clinical studies have implicated *TP53* loss as a central driver of venetoclax resistance, reinforcing the need for rational combination therapies [37–39]. Prior studies have shown that *TP53* mutations cause an inferior response to chemotherapy and poor survival in AML and other leukemias. Most have focused on the hypothesis that p53 dysfunction impairs apoptosis through defective transactivation of pro-apoptotic genes. However, our findings suggest that this model is incomplete.

Canonically, p53 initiates apoptosis by transcriptionally activated BH3-only proteins that trigger mitochondrial outer membrane permeabilization (MOMP), leading to downstream executioner caspase activation and cell death. In *TP53* wild-type AML, transcriptional p53 signaling is robustly activated following VenAza treatment. While this phenomenon is also observed in *TP53* mutant AML, induction of p53 signaling is much lower. This correlates with recent findings, where *TP53* deficient cells lacked feedforward signaling to trigger mitochondrial outer membrane permeabilization (MOMP) [27]. Although the field has acknowledged that MOMP is not always a definitive ‘point-of-no-return,’ its specific relevance to *TP53* mutant AML has remained unexplored until now. We demonstrate that drug-induced MOMP occurs in leukemia cells harboring *TP53* mutants that abolish transactivation function, suggesting that key regulatory checkpoints exist downstream of MOMP. These findings challenge the prevailing view of p53 as an initiator of apoptosis and instead reveal a previously unrecognized role for functional p53 in sustaining post-MOMP apoptotic signaling.

Beyond this role, we observed that *wild-type* p53 is required to sustain post-MOMP apoptosome activity, effector caspase activation, and completion of the apoptotic program in response to VenAza. Prior studies have implied that caspase-9 activity is not solely determined by cleavage status [34, 36]; rather, it depends critically on sustained apoptosome assembly, cofactor availability, and the kinetics of caspase-9 association with and dissociation from the Apaf-1 complex. The apoptosome functions as a proteolytic molecular timer in which processed caspase-9 retains activity only while bound to the complex, and this activity is subject to multiple regulatory inputs independent of cleavage. It is therefore consistent with established biochemistry that caspase-9 undergoes equivalent proteolytic processing in both *TP53* wild-type and *TP53* mutant/knockout cells, yet retains substantially lower catalytic activity in the latter.

By redefining the classical ‘point-of-no-return’ in apoptosis, we show that MOMP is not sufficient for cell death in the absence of functional *TP53*. Importantly, this post-MOMP apoptotic blockade was conserved across multiple isogenic *TP53* missense mutants, indicating a shared downstream vulnerability in *TP53* mutant AML. While previous efforts have focused on *TP53* deletions or on reactivating wild-type *TP53* function [17, 40], there is emerging evidence that *TP53* mutations may show distinct phenotypes and clinical outcomes [41]. Nevertheless, our data suggests that resistance mechanisms persist independently of upstream transcriptional regulation, requiring alternative strategies to restore apoptotic competence.

Collectively, our findings shift the paradigm of apoptotic resistance in *TP53* mutant AML. Instead of a defect in mitochondrial priming, *TP53* mutant cells have conserved post-mitochondrial failure in caspase activation. This has critical implications for biomarker-driven patient stratification and therapeutic design. Moreover, it raises new mechanistic questions about how *TP53* mutants impair executioner caspase activity – whether via defective apoptosome assembly, altered expression of endogenous caspase inhibitors, or impaired SMAC release. Understanding these mechanisms is essential for developing future therapies that can restore apoptosis and improve outcomes in this high-risk subset. Ultimately, our work identifies a “molecular bottleneck” in apoptosis execution that represents a targetable vulnerability in *TP53* mutant AML.

## Supplementary information

Supplemental Figure 1

Supplemental Figure 2

Supplemental Figure 3

Supplemental Figure 4

Supplemental Figure 5

Supplemental Figure 6

Supplemental Text

Supplemental Table

Uncropped blots

## Data Availability

The RNA-sequencing datasets generated during this study have been deposited in the NCBI Gene Expression Omnibus (GEO) under accession number GSE284469 and are currently available to reviewers through a private access token. The datasets will be made publicly available upon publication. All other data supporting the findings of this study are available from the corresponding author upon reasonable request.
