# Supplementary material for: TP53 mutations drive therapy resistance through post-mitochondrial caspase blockade
Source: Cell Death Dis. 2026 Jul 28;17(1):902. doi: 10.1038/s41419-026-09087-0 (PMC13691125; doi:10.1038/s41419-026-09087-0)
Supplement: Supplementary file 1 [file 41419_2026_9087_MOESM1_ESM.pdf]

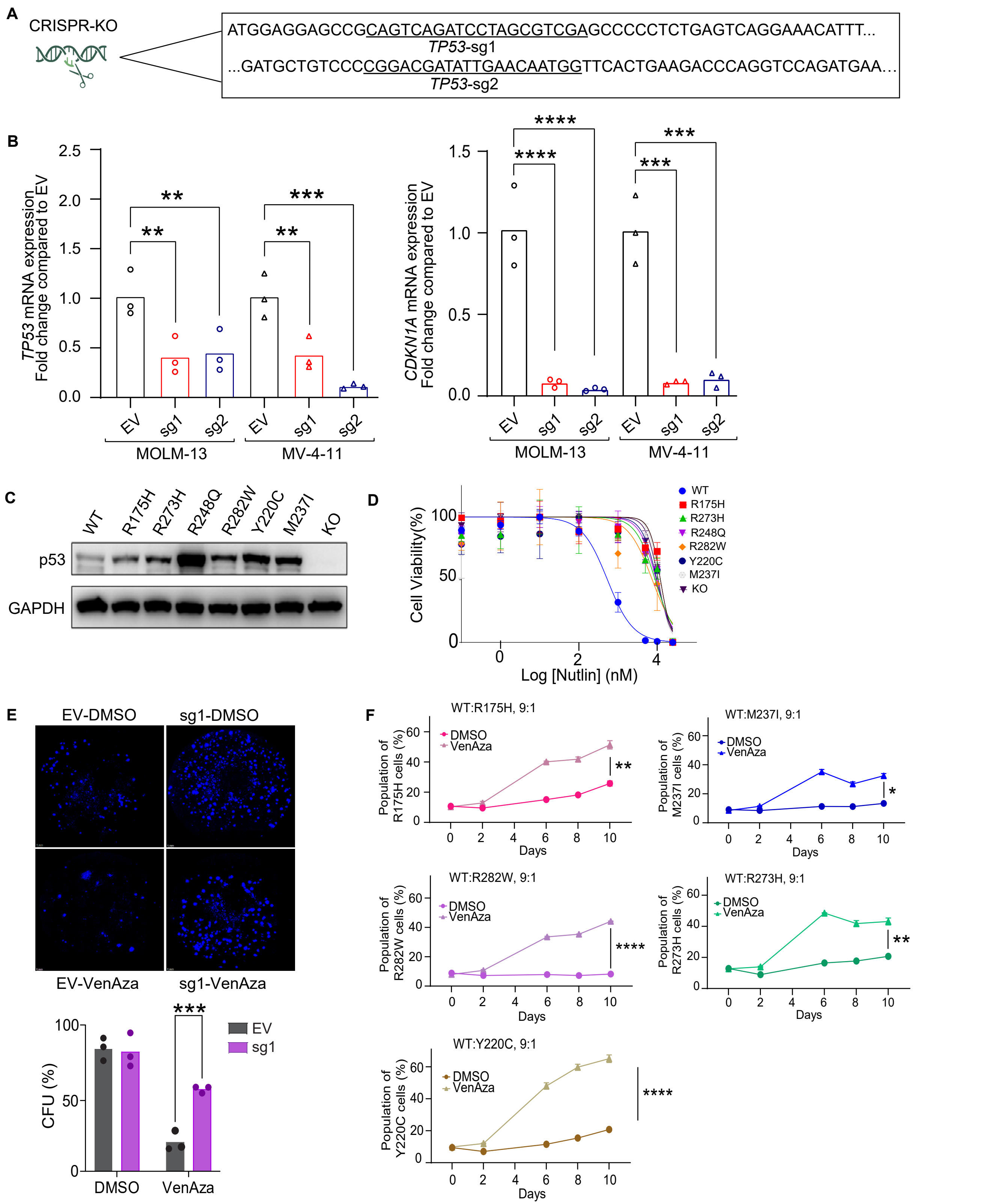

A
CRISPR-KO
ATGGAGGAGCCGCAGTCAGATCCTAGCGTCGAGCCCCCTCTGAGTCAGGAAACATTT...
TP53-sg1
...GATGCTGTCCCCGGACGATATTGAACAATGGTTCACTGAAGACCCAGGTCCAGATGAA...
TP53-sg2
B
TP53 mRNA expression Fold change compared to EV
EV
sg1
sg2
MOLM-13
MV-4-11
CDKN1A mRNA expression Fold change compared to EV
C
WT
R175H
R273H
R248Q
R282W
Y220C
M237I
KO
p53
GAPDH
D
Cell Viability(%)
Log [Nutlin] (nM)
E
EV-DMSO
sg1-DMSO
EV-VenAza
sg1-VenAza
CFU (%)
DMSO
VenAza
F
WT:R175H, 9:1
Population of R175H cells (%)
Days
WT:M237I, 9:1
Population of M237I cells (%)
WT:R282W, 9:1
Population of R282W cells (%)
WT:R273H, 9:1
Population of R273H cells (%)
WT:Y220C, 9:1
Population of Y220C cells (%)
