# Supplementary material for: TP53 mutations drive therapy resistance through post-mitochondrial caspase blockade
Source: Cell Death Dis. 2026 Jul 28;17(1):902. doi: 10.1038/s41419-026-09087-0 (PMC13691125; doi:10.1038/s41419-026-09087-0)
Supplement: Supplementary file 2 [file 41419_2026_9087_MOESM2_ESM.pdf]

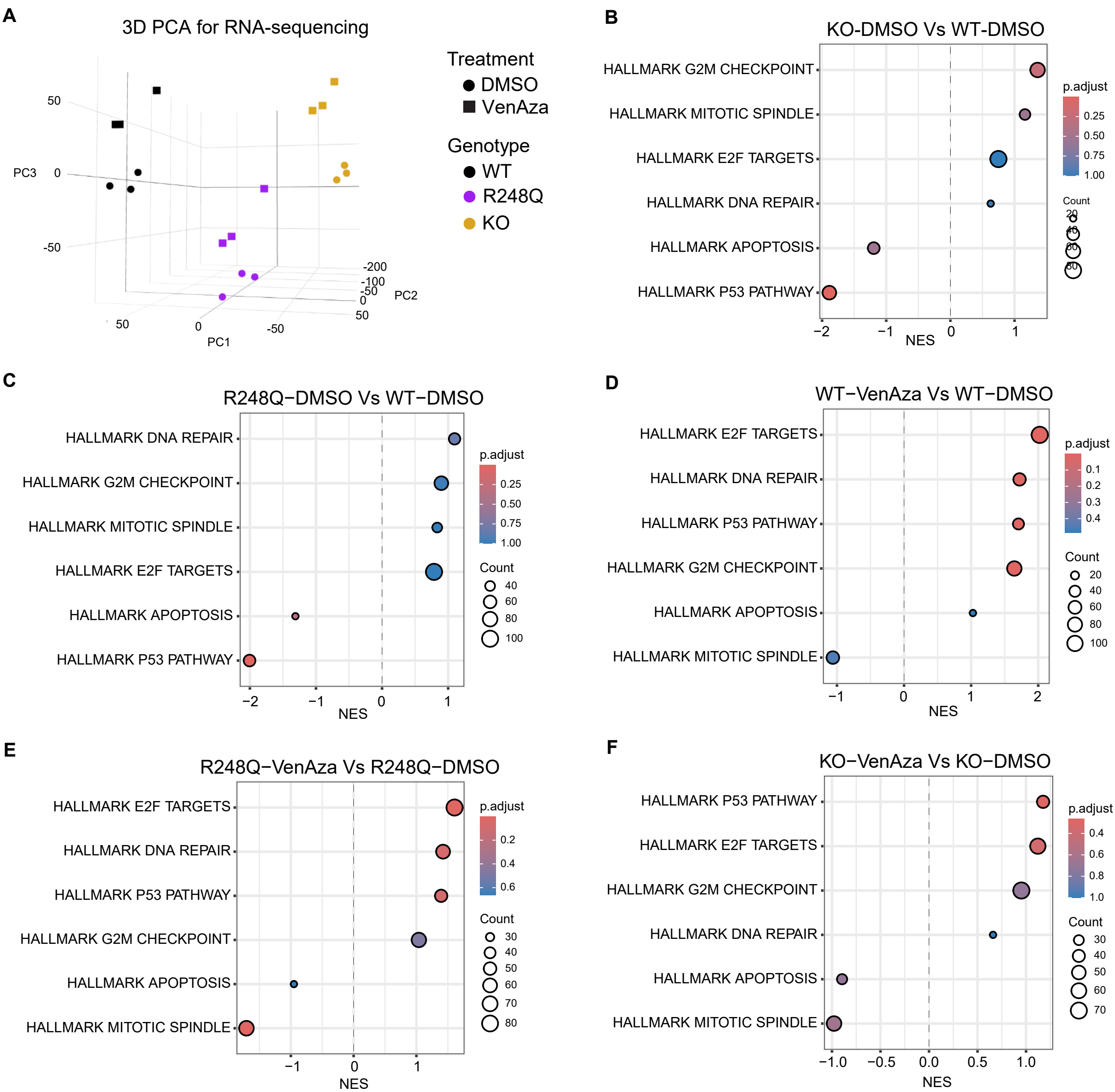

A
3D PCA for RNA-sequencing
Treatment
DMSO
VenAza
Genotype
WT
R248Q
KO
PC1
PC2
PC3
B
KO-DMSO Vs WT-DMSO
HALLMARK G2M CHECKPOINT
HALLMARK MITOTIC SPINDLE
HALLMARK E2F TARGETS
HALLMARK DNA REPAIR
HALLMARK APOPTOSIS
HALLMARK P53 PATHWAY
NES
p.adjust
Count
C
R248Q−DMSO Vs WT−DMSO
HALLMARK DNA REPAIR
HALLMARK G2M CHECKPOINT
HALLMARK MITOTIC SPINDLE
HALLMARK E2F TARGETS
HALLMARK APOPTOSIS
HALLMARK P53 PATHWAY
NES
p.adjust
Count
D
WT−VenAza Vs WT−DMSO
HALLMARK E2F TARGETS
HALLMARK DNA REPAIR
HALLMARK P53 PATHWAY
HALLMARK G2M CHECKPOINT
HALLMARK APOPTOSIS
HALLMARK MITOTIC SPINDLE
NES
p.adjust
Count
E
R248Q−VenAza Vs R248Q−DMSO
HALLMARK E2F TARGETS
HALLMARK DNA REPAIR
HALLMARK P53 PATHWAY
HALLMARK G2M CHECKPOINT
HALLMARK APOPTOSIS
HALLMARK MITOTIC SPINDLE
NES
p.adjust
Count
F
KO−VenAza Vs KO−DMSO
HALLMARK P53 PATHWAY
HALLMARK E2F TARGETS
HALLMARK G2M CHECKPOINT
HALLMARK DNA REPAIR
HALLMARK APOPTOSIS
HALLMARK MITOTIC SPINDLE
NES
p.adjust
Count
