# Supplementary material for: TP53 mutations drive therapy resistance through post-mitochondrial caspase blockade
Source: Cell Death Dis. 2026 Jul 28;17(1):902. doi: 10.1038/s41419-026-09087-0 (PMC13691125; doi:10.1038/s41419-026-09087-0)
Supplement: Supplementary file 3 [file 41419_2026_9087_MOESM3_ESM.pdf]

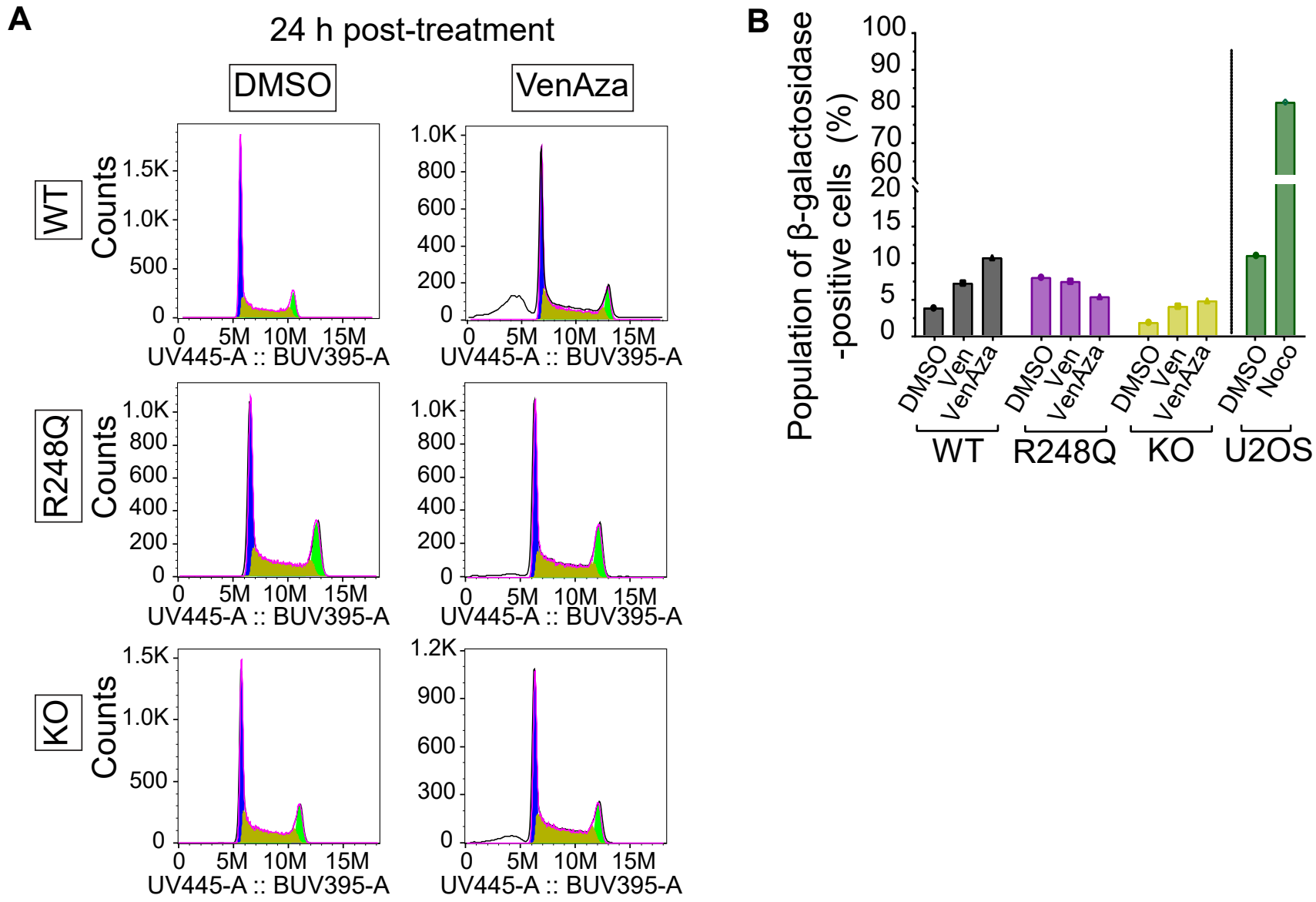
A
24 h post-treatment
DMSO
VenAza
WT
R248Q
KO
Counts
UV445-A :: BUV395-A
0
5M
10M
15M
B
Population of β-galactosidase -positive cells (%)
100
90
80
70
60
20
15
10
5
0
DMSO
Ven
VenAza
Noco
WT
R248Q
KO
U2OS
