# Supplementary material for: TP53 mutations drive therapy resistance through post-mitochondrial caspase blockade
Source: Cell Death Dis. 2026 Jul 28;17(1):902. doi: 10.1038/s41419-026-09087-0 (PMC13691125; doi:10.1038/s41419-026-09087-0)
Supplement: Supplementary file 4 [file 41419_2026_9087_MOESM4_ESM.pdf]

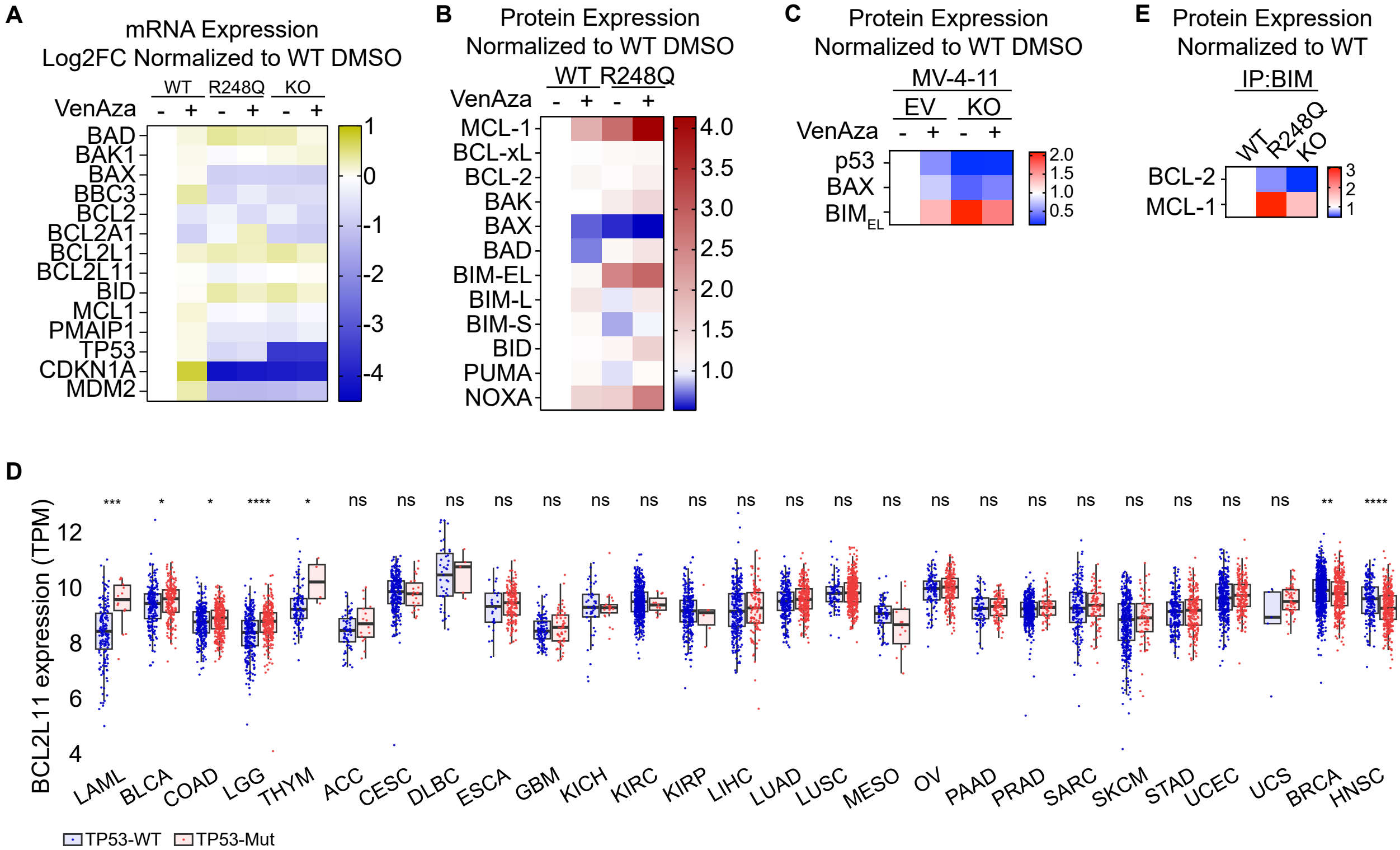
A
mRNA Expression
Log2FC Normalized to WT DMSO
WT
R248Q
KO
VenAza
BAD
BAK1
BAX
BBC3
BCL2
BCL2A1
BCL2L1
BCL2L11
BID
MCL1
PMAIP1
TP53
CDKN1A
MDM2
B
Protein Expression
Normalized to WT DMSO
WT R248Q
VenAza
MCL-1
BCL-xL
BCL-2
BAK
BAX
BAD
BIM-EL
BIM-L
BIM-S
BID
PUMA
NOXA
C
Protein Expression
Normalized to WT DMSO
MV-4-11
EV
KO
VenAza
p53
BAX
BIM$_{EL}$
E
Protein Expression
Normalized to WT
IP:BIM
WT
R248Q
KO
BCL-2
MCL-1
D
BCL2L11 expression (TPM)
LAML
BLCA
COAD
LGG
THYM
ACC
CESC
DLBC
ESCA
GBM
KICH
KIRC
KIRP
LIHC
LUAD
LUSC
MESO
OV
PAAD
PRAD
SARC
SKCM
STAD
UCEC
UCS
BRCA
HNSC
TP53-WT
TP53-Mut
