# Supplementary material for: TP53 mutations drive therapy resistance through post-mitochondrial caspase blockade
Source: Cell Death Dis. 2026 Jul 28;17(1):902. doi: 10.1038/s41419-026-09087-0 (PMC13691125; doi:10.1038/s41419-026-09087-0)
Supplement: Supplementary file 5 [file 41419_2026_9087_MOESM5_ESM.pdf]

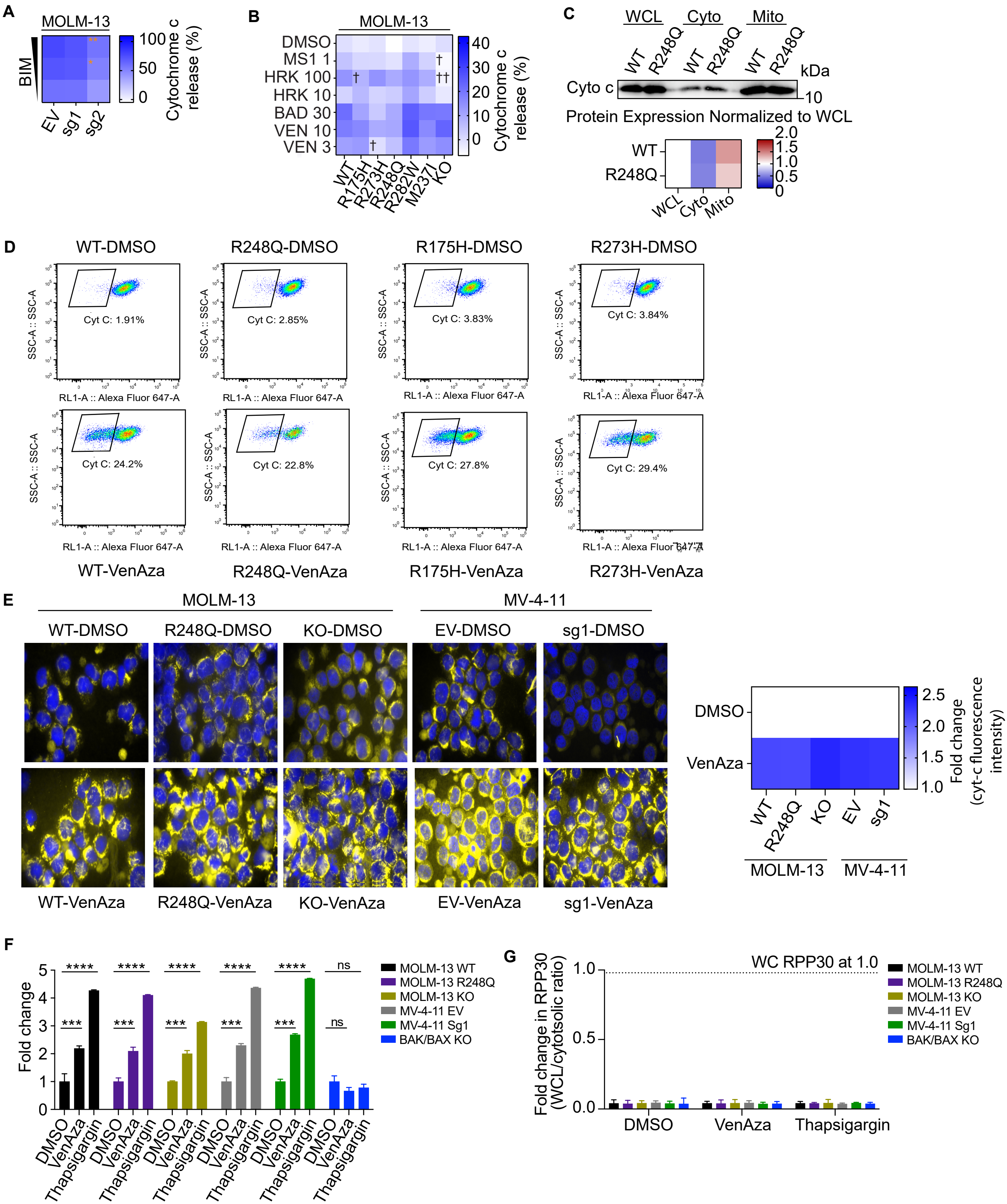
A
MOLM-13
BIM
EV
sg1
sg2
100
50
0
Cytochrome c release (%)
B
MOLM-13
DMSO
MS1 1
HRK 100
HRK 10
BAD 30
VEN 10
VEN 3
WT
R175H
R273H
R248Q
R282W
M237I
KO
40
30
20
10
0
Cytochrome c release (%)
C
WCL
Cyto
Mito
WT
R248Q
kDa
Cyto c
10
Protein Expression Normalized to WCL
WT
R248Q
WCL
Cyto
Mito
2.0
1.5
1.0
0.5
0
D
WT-DMSO
R248Q-DMSO
R175H-DMSO
R273H-DMSO
Cyt C: 1.91%
Cyt C: 2.85%
Cyt C: 3.83%
Cyt C: 3.84%
Cyt C: 24.2%
Cyt C: 22.8%
Cyt C: 27.8%
Cyt C: 29.4%
SSC-A :: SSC-A
RL1-A :: Alexa Fluor 647-A
WT-VenAza
R248Q-VenAza
R175H-VenAza
R273H-VenAza
E
MOLM-13
MV-4-11
WT-DMSO
R248Q-DMSO
KO-DMSO
EV-DMSO
sg1-DMSO
WT-VenAza
R248Q-VenAza
KO-VenAza
EV-VenAza
sg1-VenAza
DMSO
VenAza
WT
R248Q
KO
EV
sg1
MOLM-13
MV-4-11
2.5
2.0
1.5
1.0
Fold change (cyt-c fluorescence intensity)
F
Fold change
5
4
3
2
1
0
****
***
ns
DMSO
VenAza
Thapsigargin
MOLM-13 WT
MOLM-13 R248Q
MOLM-13 KO
MV-4-11 EV
MV-4-11 Sg1
BAK/BAX KO
G
WC RPP30 at 1.0
Fold change in RPP30 (WCL/cytotsolic ratio)
1.0
0.5
0.0
DMSO
VenAza
Thapsigargin
MOLM-13 WT
MOLM-13 R248Q
MOLM-13 KO
MV-4-11 EV
MV-4-11 Sg1
BAK/BAX KO
