# Supplementary material for: TP53 mutations drive therapy resistance through post-mitochondrial caspase blockade
Source: Cell Death Dis. 2026 Jul 28;17(1):902. doi: 10.1038/s41419-026-09087-0 (PMC13691125; doi:10.1038/s41419-026-09087-0)
Supplement: Supplementary file 6 [file 41419_2026_9087_MOESM6_ESM.pdf]

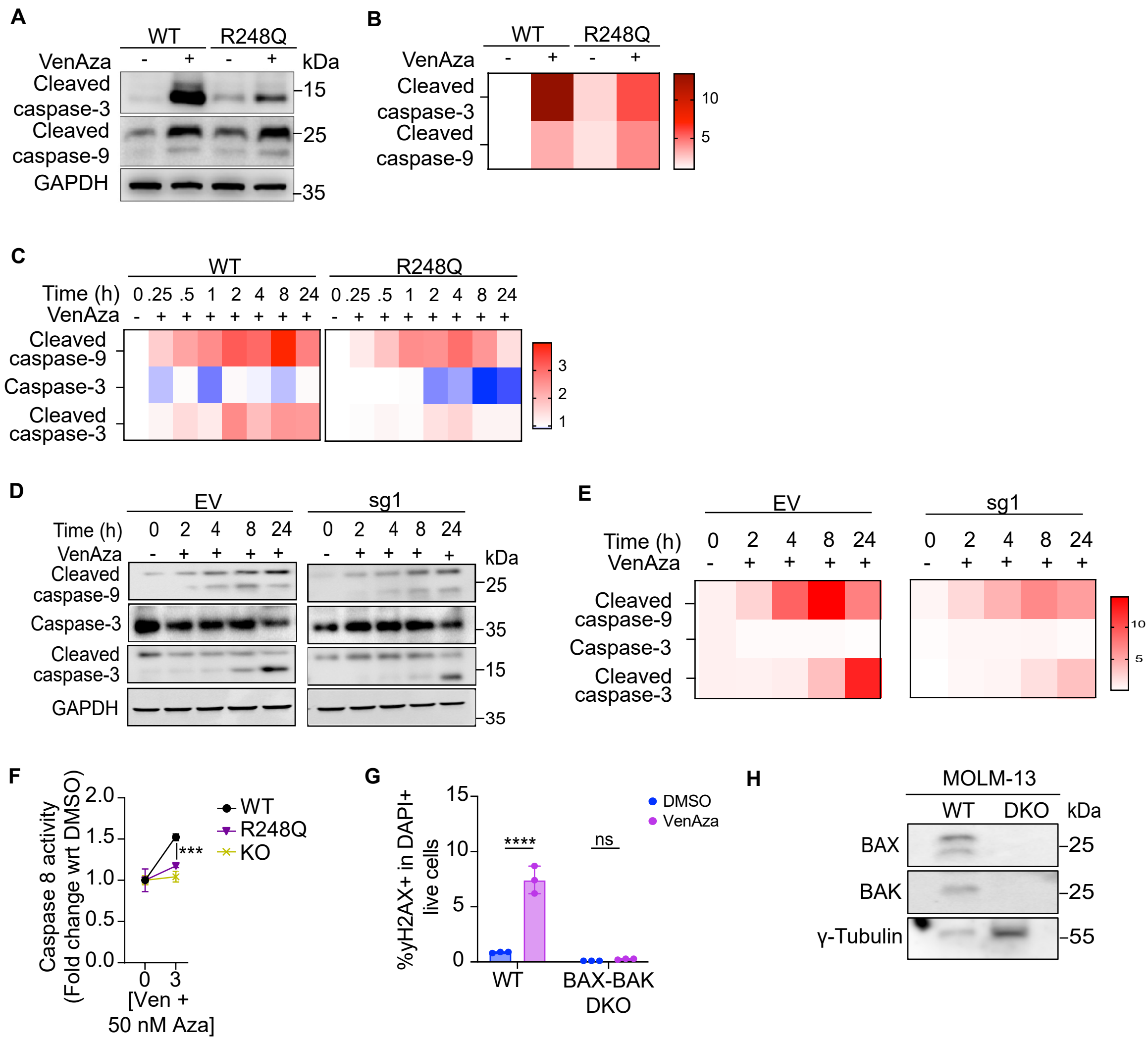

A
WT
R248Q
VenAza
kDa
Cleaved caspase-3
Cleaved caspase-9
GAPDH
15
25
35
B
WT
R248Q
VenAza
Cleaved caspase-3
Cleaved caspase-9
10
5
C
WT
R248Q
Time (h)
0 .25 .5 1 2 4 8 24
VenAza
Cleaved caspase-9
Caspase-3
Cleaved caspase-3
3
2
1
D
EV
sg1
Time (h)
0 2 4 8 24
VenAza
kDa
Cleaved caspase-9
Caspase-3
Cleaved caspase-3
GAPDH
25
35
15
E
EV
sg1
Time (h)
0 2 4 8 24
VenAza
Cleaved caspase-9
Caspase-3
Cleaved caspase-3
10
5
F
Caspase 8 activity (Fold change wrt DMSO)
2.0
1.5
1.0
0.5
0.0
0
3
[Ven + 50 nM Aza]
WT
R248Q
KO
***
G
%γH2AX+ in DAPI+ live cells
15
10
5
0
WT
BAX-BAK DKO
DMSO
VenAza
****
ns
H
MOLM-13
WT
DKO
kDa
BAX
BAK
γ-Tubulin
25
25
55
