# Supplementary material for: TP53 mutations drive therapy resistance through post-mitochondrial caspase blockade
Source: Cell Death Dis. 2026 Jul 28;17(1):902. doi: 10.1038/s41419-026-09087-0 (PMC13691125; doi:10.1038/s41419-026-09087-0)
Supplement: Supplementary file 7 [file 41419_2026_9087_MOESM7_ESM.docx]

**SUPPLEMENTAL METHODS**

**Cell cycle and cell death studies**

To determine cell cycle changes within a cell line, 5.0×10^5^ cells were permeabilized and fixed using 70% ice cold ethanol and maintained at 4 ^°^C. Next, cells were washed with PBS, stained with 400 µL of DAPI with 0.1% Triton-X, and incubated for 20 minutes. Fixed cells were analyzed using flow cytometry (LSRFortessa X-20) at an ultraviolet wavelength of 496 nM. To measure cell death, triplicates of 1.0×10^4^ cells were seeded in the 96-well plate with either DMSO (control) or the drug of interest and incubated at 37°C for the treatment duration. Cells were stained with annexin V and propidium iodide solutions 10 minutes prior to acquisition on Attune NxT Flow Cytometer. Data was analyzed on FlowJo.

**Cytotoxicity assay**

To determine viability of cells, 1.0×10^4^ cells were treated with corresponding agents at various concentrations in 96-well plates and incubated for 72 hours at 37°C. CellTiter-Glo (Promega) was added in a volume ratio of 4:1 (cell culture media:work solution), vigorously shaken for 2 minutes, and incubated for 10 minutes at room temperature. Plates were protected from light before the acquisition of luminescence signaling using a Hidex sense multi-plate reader. Absolute viability was converted to percentage viability vs. DMSO control treatment. Subsequently, IC50 values and a nonlinear fit of log (inhibitor) vs. normalized response was obtained using GraphPad Prism.

**Western blot and coimmunoprecipitation**

Cells were lysed in NP-40 lysis buffer (150 mM NaCl, 1% NP-40, 50 mM Tris-Cl pH 8.0) for coimmunoprecipitation or cell lysis buffer (Cell Signaling Technology #9803) for western blot supplemented with protease inhibitor and phosphatase inhibitor (when needed) for 45 minutes at 4°C according to the manufacturer’s protocol. Mitochondria were isolated using the Mitochondria Isolation Kit (ThermoFisher). Protein concentration was determined using Pierce BCA Protein Assay Kit (ThermoFisher). For western blot: Proteins were boiled in Laemmli Sample Buffer (Bio-Rad) containing 10% β-mercaptoethanol (Sigma-Aldrich), separated by SDS-PAGE, and transferred onto nitrocellulose membranes. For coimmunoprecipitation: 300 μg of pre-cleared lysates from each sample was incubated with Dynabeads Protein G beads (ThermoFisher) overnight prior to incubation with BIM antibody (Cell Signaling Technology, #2933) at 4°C for 4 hours. Beads were washed with NP-40 lysis buffer and washing buffer before eluting using elution buffer from Dynabeads™ Protein G Immunoprecipitation Kit (ThermoFisher). For both: Membranes were blocked for 1 hour with 5% milk (Bio-Rad) dissolved in TBST (w/v) and incubated overnight in primary antibodies diluted in 5% milk. On the next day, membranes washed with TBST (w/v) and incubated with secondary antibody for 1 hour, then washed again with TBST (w/v) before developing the plots using SuperSignal West Femto Maximum Sensitivity Substrate (ThermoFischer). List of antibodies used are listed in Supplemental Table 2. For comparison between blots in Figure 6C and S6A, images were acquired simultaneously.

**Immunofluorescence analysis for cytochrome c release**

MOLM-13 and MV-4-11 cells were seeded at 0.4 × 10^6^ cells/mL (4 mL per well in 6-well plates) and treated with the indicated compounds for 24 h. Following treatment, cells were collected, washed once with cold PBS, and resuspended in 1 mL PBS on ice. 50μL of cell suspension was loaded onto glass slides and cytospun at 1000 rpm for 5 minutes. Cells were immediately fixed in pre-chilled methanol for 10 minutes and subsequently washed 3 times with PBS. Cells were permeabilized in 0.1–0.5% Triton X-100 in PBS for 10–20 minutes at room temperature, followed by PBS washing. Slides were blocked in PBS containing 5% BSA and 0.1% Tween-20 for 20 minutes at room temperature. Primary antibodies and fluorescent dyes were diluted in blocking buffer and incubated for 40 minutes at room temperature in the dark. After washing with PBS, slides were mounted using anti-fade mounting medium and sealed. Images were acquired using an Olympus APEXVIEW APX100 system with CellSens software. Quantification of cytochrome c release was performed using ImageJ.

**mtDNA Quantification**

Cells were harvested and washed twice with ice-cold PBS. Cytosolic DNA was isolated using a digitonin-based fractionation method as previously described. Briefly, cell pellets were resuspended in digitonin lysis buffer (150 mM NaCl, 50 mM HEPES, pH 7.2–7.5, 0.002% digitonin) and incubated on ice for 10 minutes to selectively permeabilize the plasma membrane while preserving mitochondrial integrity. Lysates were centrifuged at 1,000 × g for 3 minutes at 4°C to remove nuclei and intact cells, and the supernatant was further centrifuged at 17,000 × g for 10 minutes at 4°C to eliminate mitochondria and cellular debris. The resulting supernatant was collected as the cytosolic fraction. In parallel, whole-cell lysates were processed for total DNA isolation. DNA from whole-cell and cytosolic fractions was purified using silica spin-column–based kits according to the manufacturer’s instructions. The primers used were designed according to NCBI sequences using Prime 3.0 software v.4.1.0 (Whitehead Institute, USA). The primer sequence for mtDNA1 was forward primer 5’- CCCTAAAACCCGCCACATCT -3’ and reverse primer 5’-GAGCGATGGTGAGAGCTAAGGT -3’, for RPP30 forward primer was 5'- AGATTTGGACCTGCGAGCG -3' and reverse primer 5'- GAGCGGCTGTCTCCACAAGT -3', and for 18S forward primer was 5'- TAGAGGGACAAGTGGCGTTC -3' and reverse primer 5'- CGCTGAGCCAGTCAGTGT -3'. Quantitative PCR was performed using PowerUp™ SYBR™ Green Master Mix (Applied Biosystems) according to the manufacturer’s instructions. Melt curve analysis was performed to confirm the specificity of amplification. Mitochondrial DNA was amplified using primers targeting mtDNA1 and normalized to 18S rDNA to control for DNA input. Nuclear DNA contamination in cytosolic fractions was assessed using primers against RPP30, and cytosolic RPP30 levels were compared to matched whole-cell samples to confirm fraction purity. Relative mtDNA abundance was calculated using the 2^^-ΔCt^ method.

**SUPPLEMENTAL FIGURE LEGENDS**

**Supplemental Figure 1.**

**(A)** Two sgRNAs used for CRISPR knockout. **(B)** Validation of CRISPR *TP53* knockout in isogenic MOLM-13 cells. **(C)** Western blot of p53 expression across *TP53*^+/+^, *TP53*^-/mut^, and *TP53*^-/-^ cells. **(D)** CellTiter-Glo assay in MOLM13 TP53*^+/+^*, TP53*^-/R248Q^*, and *^TP53-/-^* cells treated with Nutlin-3A for 48 h. **(E)** Representative MV-4-11 colony formation following 72 h exposure to DMSO or VenAza in RPMI and 9 days in MethoCult. Colonies were quantified and normalized to DMSO controls. Data are shown as mean ± SEM (n = 3). Two-way ANOVA with Tukey’s multiple comparisons test between EV and sg1. ***P < 0.001. **(F)** Clonal competition assay evaluating the competition between the cells in coculture after treatment with DMSO or VenAza (venetoclax 3 nM and azacitidine 50 nM) for 10 days.

**Supplemental Figure 2.**

**(**A) Principal component analysis (PCA) of RNA-seq data from all samples. PC1, PC2, and PC3 distinguish samples based on genotype and treatment. Genotypes are color-coded, while treatment conditions are indicated by shape. **(B-F)** Gene set enrichment analysis (GSEA) using MsigDB for pathways: **(B)** *TP53^-/-^* treated with DMSO vs *TP53*^+/+^ treated with DMSO; **(C)** *TP53^-/R248Q^* treated with DMSO vs *TP53*^+/+^ treated with DMSO; **(D)** *TP53^+/+^* treated with VenAza vs *TP53*^+/+^ treated with DMSO; **(E)** *TP53^-/R248Q^* treated with VenAza vs *TP53^-/R248Q^* treated with DMSO; **(F)** *TP53^-/-^* treated with VenAza vs *TP53^-/-^* treated with DMSO. For all figures unless otherwise specified, treatment with VenAza: venetoclax 3 nM, azacitidine 50 nM.

**Supplemental Figure 3.**

MOLM-13 *TP53*^+/+^, *TP53*^-/R248Q^, and *TP53^-/-^* cells treated with DMSO or VenAza for 24 hours, followed by **(A)** cell cycle profiles. **(B)** β-galactosidase assay in *TP53*^+/+^, *TP53*^-/R248Q^, and *TP53*^+/+^ cells treated with DMSO, venetoclax (1 nM), or VenAza (venetoclax 1 nM and azacitidine 50 nM) for 14 days and in U2OS cells treated with DMSO or nocodazole (100 ng/mL) for 4 days.

**Supplemental Figure 4.**

**(A)** Heatmap representing mRNA expression of apoptosis genes obtained from RNA-seq of isogenic MOLM-13 cells normalized to *TP53*^+/+^ cells treated with DMSO. **(B)** Heatmap of densitometry analysis with values normalized to β-Actin or GAPDH, followed by normalization to *TP53*^+/+^ DMSO, corresponding to Figure 4A. **(C)** Heatmap of densitometry analysis with values normalized to β-Actin, followed by normalization to *TP53*^+/+^ DMSO, corresponding to Figure 4B. **(D)** *BCL2L11* mRNA levels across multiple cancers from the TCGA database, stratified according to *TP53* status. Analysis via the Wilcoxon test. **(E)** Heatmap of densitometry analysis with values normalized to *TP53*^+/+^, corresponding to Figure 4D.* p<0.05, ** p<0.01, *** p<0.001, **** p<0.0001.

**Supplemental Figure 5. (A)** Baseline BH3 profiling heatmap shows mitochondrial priming response of DMSO and titrated BIM for MOLM-13 *EV* and *KO* (sg1 and sg2). BIM concentrations used: 0.03-3.0 μM. **(B)** Baseline BH3 profiling heatmap showing the mitochondrial priming response of DMSO, MS-1, HRK, BAD, and venetoclax for MOLM-13 isogenic cells; numbers representing the concentrations in μM. Values normalized to *TP53*^+/+^ DMSO. Dagger = decreased priming. Asterisk = increased priming. **(C)** Western blot of cytochrome c localization in whole cell lysate (WCL), cytoplasm (cyto), and mitochondria (mito). Heatmap of densitometry analysis with values normalized to whole cell lysate (WCL). **(D)** Flow cytometric analysis of cytochrome c (cyt c) release in MOLM-13 *TP53*^+/+^ and *TP53^-/mut^* (R273H, R248Q, R175H) cells treated with DMSO or VenAza for 24 hours. Cyt c–low cells were gated as undergoing MOMP. Percentages indicate the fraction of cyt c–low cells. **(E)** Representative images of cytochrome c localization in *TP53*^+/+^ and *TP53^-/mut^* MOLM-13 and MV-4-11 cells following 24-hour treatment with DMSO or VenAza. Cytochrome c is shown in yellow, and nuclei are counterstained with DAPI (blue). A corresponding heatmap depicts quantitative analysis of cyt c redistribution across genotypes and treatment conditions. Quantification is shown as fold change relative to corresponding DMSO-treated cells (right). **(F)** Quantification of cytosolic mtDNA (mtDNA/18S rDNA) in MOLM-13 and MV-4-11 cells following treatment. Cytosolic mtDNA was measured by qPCR and normalized to respective DMSO controls. *TP53*^+/+^ and *TP53^-/mut^* cells show comparable induction of cytosolic mtDNA, indicating intact and similar MOMP across genotypes. In contrast, BAX/BAK double knockout cells exhibit markedly reduced mtDNA release. Data represent mean ± SEM (n = 3). Two-way analysis of variance (ANOVA) was performed between DMSO vs VenAza and DMSO vs Thapsigargin across genotypes, followed by Tukey’s multiple comparisons test. P values are indicated. ****P < 0.0001 and ns. **(G)** qPCR quantification of nuclear DNA (RPP30) in cytosolic and whole-cell fractions from MOLM-13 and MV-4-11 cells following DMSO or VenAza treatment. RPP30 levels were measured to assess nuclear contamination and fraction purity.

**Supplemental Figure 6**

**(A)** Western blot and **(B)** densitometry of cleaved caspase-9, cleaved caspase-3 and total caspase-3 after 24 hours VenAza treatment of MOLM-13 *TP53*^+/+^ and *TP53*^-/R248Q^, run on the same gel. **(C)** Heatmap of densitometry analysis with values normalized to β-Actin or GAPDH, followed by normalization to *TP53*^+/+^ DMSO, corresponding to Figure 6C. **(D)** Immunoblot analysis of cleaved caspase-9, caspase-3, and cleaved caspase-3 in MV-4-11 *EV* and *sg1* cells treated with VenAza for the indicated time points (0–24 hours). **(E)** Heatmap showing densitometric quantification of the immunoblot. **(F)** Caspase-8 activity after treatment with VenAza. **(G)** Quantification of γH2AX⁺ cells in TP53^+/+^ and BAX-BAK double knockout (DKO) MOLM-13 cells treated with DMSO or VenAza. Percent γH2AX⁺ cells were determined by flow cytometry. Data are shown as mean ± SEM (n = 3). Statistical analysis was performed using two-way ANOVA with Bonferroni correction for multiple comparisons. ****P < 0.0001; ns **(H)** Western blot confirming BAX/BAK double knockout (DKO) in MOLM-13 cells. For all figures unless otherwise specified, treatment with VenAza: venetoclax 3 nM, azacitidine 50 nM.

**SUPPLEMENTAL TABLES**

**Supplemental Table 1.** sgRNA sequences used for CRISPR/Cas9-mediated knockout of BIRC5, XIAP, and TP53 (two independent guide RNAs).

**Supplemental Table 2.** Antibodies used for immunoblotting, including target, supplier, and catalog number.
