# Supplementary material for: TP53 mutations drive therapy resistance through post-mitochondrial caspase blockade
Source: Cell Death Dis. 2026 Jul 28;17(1):902. doi: 10.1038/s41419-026-09087-0 (PMC13691125; doi:10.1038/s41419-026-09087-0)
Supplement: Supplementary file 8 [file 41419_2026_9087_MOESM8_ESM.docx]

**SUPPLEMENTAL TABLES**

**Supplemental Table 1: sgRNA sequence used for generation of** knockout cells.

| **Target gene** | **sgRNA sequence** |
| --- | --- |
| TP53 (sg1) | TCGACGCTAGGATCTGACTG |
| TP53 (sg2) | CCATTGTTCAATATCGTCCG |

**Supplemental Table 2: Antibodies used for immunoblotting.**

| **Antibody** | **Source** | **Catalog No.** |
| --- | --- | --- |
| p53 Antibody (DO-1) | Santa Cruz Biotechnology | sc-126 |
| Cleaved Caspase-3 (Asp175) Antibody | Cell Signaling Technology | #9661 |
| Cytochrome c Antibody (A-8) | Santa Cruz Biotechnology | sc-13156 |
| Caspase-3 Antibody | Cell Signaling Technology | #9662 |
| GAPDH (D16H11) XP® Rabbit mAb | Cell Signaling Technology | #5174 |
| Bcl-xL (54H6) Rabbit mAb | Cell Signaling Technology | #2764 |
| Mcl-1 (D2W9E) Rabbit mAb | Cell Signaling Technology | #94296 |
| Bcl-2 (124) Mouse mAb | Cell Signaling Technology | #15071 |
| Bak (D4E4) Rabbit mAb | Cell Signaling Technology | #12105 |
| Bax Antibody | Cell Signaling Technology | #2772 |
| Bad (D24A9) Rabbit mAb | Cell Signaling Technology | #9239 |
| Bim (C34C5) Rabbit mAb | Cell Signaling Technology | #2933 |
| BID Antibody | Cell Signaling Technology | #2002 |
| Noxa (D8L7U) Rabbit mAb | Cell Signaling Technology | #14766 |
| Puma (D30C10) Rabbit mAb | Cell Signaling Technology | #12450 |
| Phospho-Histone H2A.X (Ser139) (20E3) Rabbit mAb | Cell Signaling Technology | #9718 |
| Cleaved Caspase-9 (Asp330) (D2D4) Rabbit mAb | Cell Signaling Technology | #7237 |
| β-Actin (8H10D10) Mouse mAb | Cell Signaling Technology | #3700 |
| PARP Antibody | Cell Signaling Technology | #9542 |
| Apaf-1 (R205) Antibody | Cell Signaling Technology | #5088 |
